# Harnessing pyroptosis in breast cancer therapy: immunological mechanisms and emerging biomaterial strategies

**DOI:** 10.1038/s41420-026-02996-1

**Published:** 2026-03-12

**Authors:** Richmond Kwame Frimpong Asiedu, Mahamadou Souley Abdou, Rongbin Wei, Zhongdang Xiao, Bing Li, Ming Hu

**Affiliations:** 1 Jiangsu Key Laboratory of Marine Pharmaceutical Compound Screening, College of Pharmacy, Jiangsu Ocean University, Lianyungang, China; 2 State Key Laboratory of Bioelectronics, National Demonstration Center for Experimental Biomedical Engineering Education, School of Biological Science and Medical Engineering, Southeast University, Nanjing, China; 3 Jiangsu Institute of Marine Resources Development, Jiangsu Ocean University, Lianyungang, China; 4 Department of Hematology, Tongji Hospital, Tongji University School of Medicine, Tongji University, Shanghai, China; 5 Medical Department, Donghai Hospital Affiliated to Kangda Medical College of Nanjing Medical University, Lianyungang Donghai People’s Hospital, Lianyungang, China

**Keywords:** Breast cancer, Cell death

## Abstract

Breast cancer is a complex and predominant long-term medical condition in women. Its complexity is related to its diversity, variable clinical outcomes, and resistance to standard treatments, emphasizing the need for novel treatment strategies. Pyroptosis is a lytic and inflammatory programmed cell death regulated by the gasdermin protein family. It is a critical factor in cancer biology and plays distinct roles in tumor initiation, progression, and response to anticancer treatments. Pyroptosis, with its inflammatory properties, involves the formation of pores in the plasma membrane, resulting in cellular swelling, lysis, and the release of pro-inflammatory intracellular contents, thereby producing a robust immune response. Emerging studies suggest that pyroptosis plays a crucial role in breast cancer development, indicating its potential as a target for novel therapeutic strategies in breast cancer. Pyroptosis has the potential to directly kill malignant cells and elicit anti-cancer immunity. Therefore, targeting pyroptosis is a promising strategy for cancer therapy development. This review compiles recent advancements in the understanding of pyroptosis as a therapeutic target in breast cancer and discusses the molecular pathways responsible for regulating pyroptosis, the expression and roles of gasdermin proteins in breast cancer, and the modulation of pyroptosis to improve anticancer efficacy and address drug resistance.

## Facts

- Pyroptosis, a type of programmed inflammatory cell death, has emerged as a potential therapeutic target in breast cancer.
- Studies have indicated that gasdermin may promote cancer cell death and modulate the immune response.
- Certain chemotherapeutic agents and natural compounds have been shown to facilitate pyroptosis in breast cancer cells, suggesting potential therapeutic strategies for breast cancer treatment.
- A major obstacle in the therapeutic application of pyroptosis is its ability to selectively induce apoptosis in cancer cells without causing toxicity to normal tissues, which remains an active area of investigation.

## Open questions

- How do specific TME components affect pyroptosis and antitumor immunity in breast cancer?
- How can pyroptosis-induced inflammation be optimized to enhance the efficacy of immunotherapy in breast cancer?
- What are the key resistance mechanisms to pyroptosis, and how can combination therapies be used to overcome these mechanisms?
- Which biomarkers can predict the response to pyroptosis-inducing therapies, and how can they improve the design of clinical trials?
- How does genetic and phenotypic tumor heterogeneity affect the efficacy of pyroptosis-inducing therapy?

## Introduction

Breast cancer (BC) remains one of the most prevalent malignancies among women globally, with 2.3 million new cases and 685,000 deaths reported in 2020 [1]. Despite advances in detection and treatment, the prognosis of many patients, particularly those with advanced or treatment-resistant disease, remains poor. Conventional therapies, such as surgical resection, radiotherapy, and chemotherapy, have improved survival rates. However, the heterogeneous nature of breast cancer and drug resistance continue to pose major challenges [1]. Pyroptosis is a programmed inflammatory cell death that involves cell swelling, membrane rupture, and the release of pro-inflammatory substances [2]. This process differs from other cell death pathways and shows promise for breast cancer treatment. In contrast to apoptosis, which is immunologically silent, pyroptosis stimulates robust antitumor immune response [3]. Pyroptosis has dual effects on BC development [1, 2]. It activates antitumor immunity by releasing proinflammatory cytokines and promoting immune cell recruitment. However, a proinflammatory microenvironment can fuel tumor growth and metastasis [2]. Understanding the interactions between pyroptosis and the tumor microenvironment in breast cancer is crucial for developing effective treatment strategies. By elucidating these molecular mechanisms, particularly the role of the gasdermin protein family, researchers can induce pyroptosis in cancer cells while minimizing toxicity to normal tissues [2]. The combination of pyroptosis-inducing agents with therapies, such as immune checkpoint inhibitors, may enhance antitumor immunity and overcome resistance [1, 3]. This review examines recent research on pyroptosis in breast cancer therapy, focusing on molecular pathways, gasdermin proteins, and strategies to improve treatment outcomes.

## Breast cancer: distinctive features among solid tumors

Compared with many other solid tumors, breast cancer has distinct characteristics in terms of its pathological features, pathogenesis, tumor microenvironment, immune landscape, and mechanisms of drug resistance. These differences are crucial for a comprehensive understanding of the disease and for developing targeted therapeutic strategies.

a. Pathological Features

Breast cancer exhibits significant pathological heterogeneity, with various histological types and molecular subtypes, such as Luminal A, Luminal B, HER2-positive, and Triple-Negative Breast Cancer (TNBC) [4]. This diversity distinguishes it from other cancers with simpler classification systems. This differs from other cancers, which may have fewer recognized molecular subtypes or different primary classifications than breast cancer. For example, lung cancer is generally classified into small-cell and non-small-cell types, with further distinctions, such as adenocarcinoma and squamous cell carcinoma, based on cell appearance and origin [5]. The Nottingham Grading System for breast cancer assesses tubular formation, nuclear pleomorphism, and mitotic count to determine tumor grade [6], using parameters that differ from those of grading systems for other cancers. Metastasis to the breast from other primary sites is also a pathological consideration, necessitating an accurate differential diagnosis based on the origin [7].

b. Pathogenesis

The molecular basis of breast cancer development is often unique to each patient. A substantial proportion of breast cancers are characterized by hormone receptor positivity and/or HER2 amplification, which guide specific targeted therapies [8]. Although genetic mutations are common in all cancers, the prevalence of specific driver mutations varies among different types of cancer. For instance, mutations in BRCA1 and BRCA2 significantly increase the risk of breast and ovarian cancers, highlighting distinct genetic predispositions [9]. Specific pathways and their activation sequences also differ; the PI3K/AKT/mTOR signaling pathway is frequently altered in breast cancer [10], but its role and downstream effects may differ from those in other cancers, in which other pathways may be more dominant [11].

c. Tumor Microenvironment

The breast cancer TME is a complex ecosystem comprising immune cells, stromal cells (e.g., cancer-associated fibroblasts and adipocytes), and the extracellular matrix [12]. Although all solid tumors interact with their microenvironment [13], the specific compositions and influences of these elements differ among different tumors. For instance, the TME of breast cancer often contains a significant number of tumor-associated macrophages and cancer-associated adipocytes, which play crucial roles in tumor progression [12]. In contrast, cancers such as pancreatic ductal adenocarcinoma are characterized by an exceedingly dense fibrous stroma that drives their aggressiveness [14]. The interplay between immune cells and stromal components in breast cancer has unique implications for its progression and response to therapy [15].

d. Immune Characteristics

Breast cancer is often considered an “immunologically colder” tumor than highly immunogenic cancers, such as melanoma [16]. This is often reflected in a lower tumor mutational burden, fewer tumor-infiltrating lymphocytes, and lower PD-1/L1 expression [16, 17]. This contrasts with cancers that respond well to immune checkpoint inhibitors, where a higher TMB and pre-existing immune infiltration often predict better outcomes [18]. Although immunotherapy has revolutionized the treatment of some solid tumors, the overall response rate in breast cancer is lower, necessitating the development of combination therapies [19]. The immune landscape of breast cancer is dynamic and heterogeneous, with subtype variations [17, 18]. Distinct immune responses related to patient ancestry have also been reported in breast cancer, showing a stronger overall immune presence and a more exhausted CD8 + T cell profile in certain populations [20].

e. Drug Resistance

Drug resistance remains a major challenge; however, the underlying mechanisms vary among cancers. In breast cancer, acquired resistance often involves the overexpression of efflux pumps, activation of compensatory signaling pathways (e.g., PI3K/AKT/mTOR pathway dysregulation [21]), and the presence of cancer stem cells (CSCs) [22]. TNBC is known for its intrinsic resistance to many conventional therapies owing to the lack of hormone receptors and HER2 amplification [23]. Although multidrug resistance is a general phenomenon across cancers [24], the specific pathways that are upregulated and the role of the tumor microenvironment in conferring resistance can differ significantly among cancers. The context-specific intrinsic resistance of TNBC is a notable feature [23]. Consequently, strategies to circumvent drug resistance in breast cancer often involve targeting specific resistance mechanisms and developing combinatorial approaches [19].

### TNBC: distinct pathological and molecular features

TNBC is a clinically significant and biologically distinct subtype characterized by the absence of estrogen, progesterone, and human epidermal growth factor receptor 2 expression. This lack of expression profoundly impacts its pathogenesis, clinical behavior, and treatment response compared with those of other breast cancer subtypes [25]. TNBC is widely recognized as an aggressive group of tumors [25] and is often associated with high-grade tumors [26].

TNBC is characterized by significant molecular heterogeneity, including distinct genetic, transcriptional, and clinical profiles [26]. These intrinsic subtypes possess unique genomic alterations and pathway-dependent characteristics. Notably, most TNBC cases exhibit a “basal-like” molecular signature [27]. Compared with other breast cancer subtypes, TNBC frequently displays mutations in TP53 and PIK3CA and is associated with BRCA1 mutation [28]. The phosphoinositide 3-kinase/protein kinase B/mechanistic target of rapamycin (PI3K/AKT/mTOR) signaling pathway is commonly overactivated in TNBC [29]. Genomic aberrations within the PI3K pathway, including PTEN loss/mutation, are observed in basal-like tumors that often overlap with TNBC [30]. This molecular diversity contributes to TNBC’s aggressive clinical course, high recurrence rates, and resistance to conventional chemotherapy of TNBC, underscoring the critical need for targeted therapies [31]. A comprehensive understanding of these intricate pathological and molecular distinctions is essential for developing subtype-specific therapeutic strategies [32].

## Molecular mechanisms of pyroptosis

The molecular mechanisms of pyroptosis involve a complex interplay of signaling pathways, with the gasdermin family acting as the central executor of programmed cell death [33]. These mechanisms are categorized into canonical, non-canonical, and alternative pathways, each initiated by distinct stimuli and involving different caspases [34]. Table 1 compares the principal pyroptotic cascades, highlighting their initiating triggers, core caspases, and gasdermin family members. This overview highlights that breast cancer-relevant agents, such as cisplatin and doxorubicin, engage distinct molecular executors.

**Table 1:** Pyroptosis Molecular Pathways in Breast Cancer.

Pathway	Key Triggers	Main Caspases	GSDM Family Involved	Mechanism	Ref
Canonical	Inflammasomes (NLRP1, NLRP3, NLRC4, AIM2, Pyrin)	Caspase-1	GSDMD	Inflammasome-activated caspase-1 cleaves GSDMD; N-terminal forms membrane pores, inducing cell lysis	[215]
Non-canonical	Cytosolic LPS (bacterial infection)	Caspase-4/5 (human), Caspase-11 (mouse)	GSDMD	Caspase-4/5/11 directly cleave GSDMD in response to LPS; pore formation leads to pyroptosis	[216]
Chemotherapy-induced	Chemotherapeutic agents (e.g. cisplatin)	Caspase-3	GSDME (main), others possible	Activates apoptotic caspase-3, which cleaves GSDME to switch apoptosis to pyroptosis in sensitive cells	[217]
Granzyme-mediated	NK cells, Cytotoxic T Lymphocytes (CTLs)	Granzymes (e.g., Granzyme B)	GSDMB, GSDME, GSDMC	Granzymes cleave gasdermins directly, triggering pyroptosis in target (tumor) cells	[218]
Other Pathways	Diverse stress signals, bacterial proteases	Serine proteases, other enzymes	Multiple	Alternative proteases can directly cleave and activate gasdermins, expanding pyroptosis triggers	[219]

The canonical pyroptosis pathway relies on inflammasomes, which are protein complexes that form when cells detect pathogens or damage [35]. Upon activation, inflammasome-associated caspase-1 cleaves pro-inflammatory cytokines pro-interleukin-1β and pro-interleukin-18 into their mature forms. Simultaneously, caspase-1 cleaves gasdermin D (GSDMD), a key pyroptotic executor [34]. The liberated N-terminal domain of GSDMD oligomerizes and inserts into the plasma membrane, forming large pores that compromise cellular integrity and facilitate the uncontrolled release of intracellular components, including IL-1β and IL-18 [33, 35].

In humans, the non-canonical pyroptotic pathway is initiated by the direct binding of cytosolic lipopolysaccharides to caspase-4 or caspase-5, leading to GSDMD cleavage and pore formation [36].

In addition to GSDMD, other gasdermin family members, including GSDMA, GSDMB, GSDMC, and GSDME, induce pyroptosis [34]. For example, GSDME, which is often silenced in cancer cells, can be activated by caspase-3, a key executioner caspase in apoptosis, to induce pyroptosis following death receptor stimulation or mitochondrial membrane disruption [37].

**Fig. 1:**
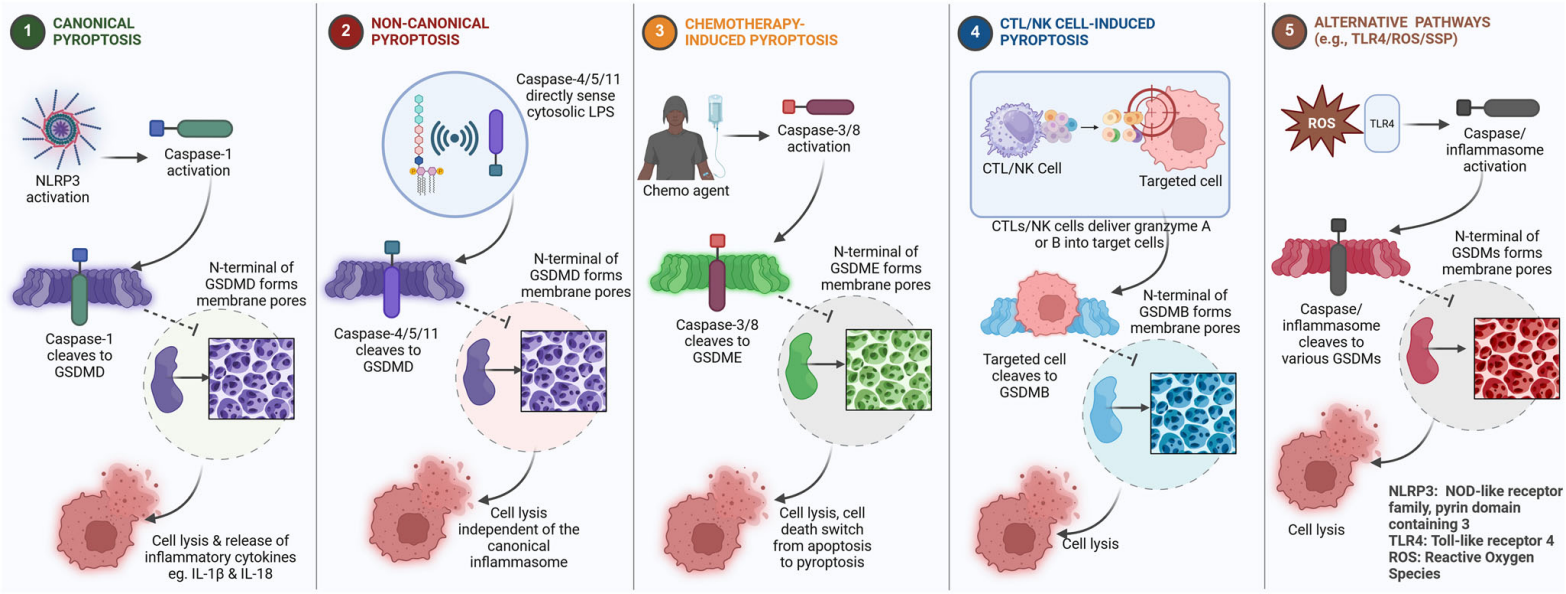
Molecular mechanism of Pyroptosis. It illustrates the molecular mechanisms underlying pyroptosis, encompassing five distinct pathways: (1) the canonical pathway involving caspase-1, NLRP3, and GSDMD; (2) the non-canonical pathway mediated by caspase-5 and GSDMD; (3) the chemotherapy-induced pathway facilitated by caspase-3 or caspase-8 and GSDME; (4) the cytotoxic T lymphocyte (CTL) and natural killer (NK) cell-driven pathway involving granzyme A or B and GSDMB; and (5) the alternative pathway, which includes factors such as TLR4, reactive oxygen species (ROS), and SSP. Each of these pathways culminates in the formation of pores within the cell membrane, resulting in cell lysis and the subsequent release of signals that enhance antitumor immune responses and modulate the tumor microenvironment.

Pyroptosis is primarily mediated by the gasdermin family of proteins. Upon activation by inflammatory caspases, the N-terminal domain of a gasdermin protein oligomerizes to form membrane pores [33]. These pores disrupt ionic gradients, leading to osmotic imbalance, cell swelling, and rupture of the cell membrane. This permeabilization also allows the release of proinflammatory cytokines and alarmins, which recruit and activate immune cells and are hallmarks of the inflammatory response associated with pyroptosis [35]. As depicted in Fig. 1, the canonical inflammasome-dependent pathway (NLRP3-caspase-1-GSDMD), non-canonical LPS-caspase-4/5/11 route, and apoptosis-associated caspase-3/GSDME pathway converge to form gasdermin pores. This schematic underscores how diverse upstream signals ultimately result in membrane rupture and the release of inflammatory cytokines, providing a mechanistic basis for therapeutic targeting.

## The gasdermin family in breast cancer

The gasdermin family, comprising GSDMA-E and PJVK, is the central executor of pyroptosis via pore-forming activity in the plasma membrane [33]. Individual members exhibit distinct roles and regulatory mechanisms in breast cancer, making them important therapeutic targets for breast cancer treatment. As detailed in Table 2, the individual gasdermins differ in their activation mechanisms and subtype associations. This comparison clarifies why targeting GSDMC may benefit TNBC, whereas restoring GSDME expression may sensitize luminal tumors to chemotherapy-induced pyroptosis.

**Table 2:** Role of gasdermin family members in the regulation of pyroptosis in breast cancer.

Gasdermin Member	Activation Mechanism	Pyroptosis Pathway	Impact on Breast Cancer	Regulatory Factors	Genetic Features	Protein Isoforms/ Structure	Subtype Association	Ref
GSDMC	Caspase-8 cleavage (induced by TNFα, nuclear PD-L1, p-Stat3)	Forms membrane pores, induces pyroptosis	High expression linked to poor prognosis and tumor necrosis	Upregulated by LINC00511/hsa-miR-573 axis; correlates with immune infiltration	Most frequently altered in breast cancer	Standard GSDM structure	Basal-like, triple-negative	[50, 61]
GSDME	Caspase-3 or granzyme B cleavage	Converts apoptosis to pyroptosis, forms membrane pores	Acts as tumor suppressor; enhances anti-tumor immunity, phagocytosis, and T/NK cell infiltration	Expression often suppressed in cancer; stabilized by USP48	Upregulated, high alteration rate	Conserved structure	All subtypes, esp. luminal-B	[54, 57]
GSDMD	Caspase-1/4/5/11 cleavage	Key executioner of pyroptosis	Involved in cancer cell death and therapy response	Regulated by non-coding RNAs (lncRNA, miRNA, siRNA)	Low mutation, methylation changes	Standard GSDM structure	All subtypes, therapy response	[220]
Other GSDMs	Less characterized in breast cancer	Potential roles in pyroptosis and tumor immunity	May influence tumor progression and immune microenvironment	Research ongoing	Rarely mutated in cancer	Lacks pore-forming activity	No established association	[39]

a. GSDMA

GSDMA remains a relatively understudied gasdermin in breast cancer. While humans have a single GSDMA gene, the GSDMA3 isoform has been reported to be upregulated in breast cancer [38]. Therapeutically, the HDAC inhibitor valproic acid has been shown to induce GSDMA-dependent pyroptosis in TNBC cells, suppress tumor growth, and enhance antitumor immunity [38]. Other approaches, such as bio-orthogonal chemical systems designed to selectively activate GSDMA, are also under preclinical investigation [39], highlighting their potential as novel therapeutic targets.

b. GSDMB

GSDMB plays a dualistic role in breast cancer that is influenced by its splice variants. For instance, the GSDMB-A isoform promotes cell proliferation and migration, whereas the GSDMB-C variant modulates the tumor microenvironment through the release of pro-inflammatory cytokines [40]. This context-dependent function underscores the complexity of GSDMB-mediated pathway regulation.

A key therapeutic mechanism involves immune effector cells; natural killer (NK) cells and chimeric antigen receptor (CAR) T-cells can cleave GSDMB, activating its pore-forming function and triggering pyroptosis specifically in GSDMB-expressing cancer cells [41]. This selective induction of cell death remodels the tumor microenvironment via the release of IL-1β and IL-18, presenting a promising strategy for immunotherapy, particularly in combination with existing modalities [40].

c. GSDMC

GSDMC is upregulated in aggressive breast cancer cell lines (e.g., MDA-MB-231) compared to normal cells, and its expression is induced by tumor microenvironment stressors, such as hypoxia [42]. GSDMC-mediated pyroptosis releases pro-inflammatory cytokines (IL-1β and IL-18), which can enhance anti-tumor immunity but may also promote tumor progression if excessive [43]. The clinical relevance of GSDMC is highlighted by its inclusion in a pyroptosis-related gene (PRG) risk model (containing GSDMC, GZMB, IL18, and TP63) that effectively stratifies breast cancer patients into high- and low-risk groups, underscoring its prognostic value [44]. Targeting GSDMC with small-molecule modulators represents a potential therapeutic strategy for influencing the tumor microenvironment [45].

Small-molecule modulators indirectly influence GSDMC activity. For instance, certain chemotherapeutic agents, such as doxorubicin, induce PD-L1 nuclear translocation, which subsequently promotes GSDMC expression [46]. Furthermore, GSDMC-mediated pyroptosis can be initiated by caspase-8 under hypoxic conditions [47], as observed in breast cancer cells, such as MDA-MB-231 [1]. This suggests that modulating hypoxic responses or caspase-8 activity may serve as an indirect strategy for GSDMC activation. While direct GSDMC inhibitors are under development, broader gasdermin pore-forming inhibitors, such as necrosulfonamide [48] and disulfiram [49], which target GSDMD, represent alternative strategies for mitigating pyroptotic effects.

Furthermore, engineered immune cells, such as CAR-T cells, represent a promising approach for selectively inducing pyroptosis in GSDMC-expressing tumor cells [44]. The association between high GSDMC levels and poor survival in breast cancer underscores its clinical significance as a therapeutic target [50].

d. GSDMD

GSDMD is a critical executor of pyroptosis [1]. Upon activation by inflammatory signals, such as those from the NLRP3 inflammasome, caspase-1 cleaves GSDMD and releases its N-terminal domain. This domain oligomerizes to form plasma membrane pores, disrupting the osmotic balance and leading to cell lysis [1]. The anti-breast cancer effects of chemotherapeutic agents, such as cisplatin, are partly mediated through the MEG3/NLRP3/caspase-1/GSDMD pathway, highlighting the therapeutic relevance of this axis [44, 51].

Enhancing GSDMD-mediated pyroptosis is a key therapeutic strategy for treating these diseases. Strategies include the development of specific pyroptosis-inducing agents, optimization of targeted drug delivery systems to tumor cells, and the use of engineered immune cells, such as CAR-T cells, to selectively trigger pyroptosis in GSDMD-expressing tumors [52, 53]. A critical focus is on identifying predictive biomarkers to stratify patients and ensure the specificity of these approaches to minimize off-target effects in normal cells [39].

e. GSDME

GSDME-mediated pyroptosis is a promising therapeutic target for breast cancer. A key mechanism involves chemotherapeutic agents, such as doxorubicin and cisplatin, which activate caspase-3 to cleave GSDME and induce pyroptosis in GSDME-expressing cells [54, 55]. This process not only directly kills tumor cells but also stimulates antitumor immunity by promoting macrophage phagocytosis and enhancing the activity of natural killer cells and CD8 + T cells [44]. This immunogenic effect provides a strong rationale for combining pyroptosis-inducing agents with immune checkpoint inhibitors, such as PD-1 blockers, to convert immunologically “cold” tumors into “hot” ones [44].

**Fig. 2:**
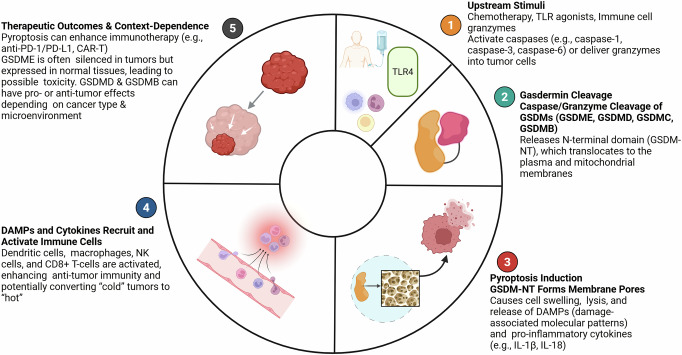
Current status of GSDM-mediated tumor therapy. (1) Chemotherapeutic agents, Toll-like receptor agonists, and immune cell granzymes activate caspases (e.g., caspase-1, -3, and-6) or deliver granzymes into tumor cells. (2) Activated caspases or granzymes cleave GSDM family members (GSDME, GSDMD, GSDMC, and GSDMB), liberating the N-terminal domain (GSDM-NT). (3) GSDM-NT is inserted into the plasma and mitochondrial membranes, forming pores that cause cell swelling, lysis, and the release of DAMPs and pro-inflammatory cytokines, such as IL-1β and IL-18. (4) The released mediators recruit and activate dendritic cells, macrophages, NK cells, and CD8⁺ T cells, thereby strengthening antitumor immunity. (5) Pyroptosis can synergize with immunotherapies (e.g., anti-PD-1/PD-L1, CAR-T) to enhance efficacy; however, GSDME expression in normal tissues and the dual roles of GSDMD/GSDMB underscore the need for selective, context-specific activation to avoid toxicity or tumor-promoting inflammation.

The efficacy of this strategy is contingent on GSDME expression, which is frequently silenced via methylation in breast cancer, an alteration associated with lymph node metastasis and poor prognosis [44, 56]. Therefore, profiling GSDME expression is critical for identifying patients who are most likely to benefit from therapies that engage the caspase-3/GSDME pathway, thereby enabling a more precise and effective treatment approach. As shown in Fig. 2, GSDM-mediated tumor therapy proceeds through multiple steps, starting with upstream stimuli such as chemotherapeutic drugs, TLR agonists, or granzymes. These activate caspases or granzymes that cleave gasdermins (GSDME, GSDMD, GSDMC, and GSDMB), releasing N-terminal fragments. The fragments form membrane pores, causing pyroptotic cell lysis and the release of DAMPs and cytokines (IL-1β and IL-18). These mediators recruit dendritic cells, macrophages, and T cells, enhancing antitumor immunity and potentially converting “cold” tumors to “hot” tumors. While transient activation enhances the efficacy of immunotherapy, excessive pyroptosis may trigger inflammation-related toxicity or tumor-promoting effects.

### GSDMs in breast cancer: differences in genes, structure, and function across subtypes

Gasdermin (GSDM) family members exhibit subtype-specific genetic alterations, structural diversity, and functional roles in breast cancer, contributing to distinct pathological behaviors.

#### Genetic information and subtype-specific alterations

GSDM genes display subtype-specific mutation and expression patterns. The luminal B subtype harbors the most diverse GSDM mutations, with GSDMB, GSDMC, and GSDMD serving as independent prognostic factors [57]. GSDMC is the most frequently altered gene, and its alterations, along with those in GSDMD and GSDMB, are associated with poor overall survival [50]. Distinct methylation patterns of GSDM genes across subtypes further highlight their potential as biomarkers [58].

#### Protein structure and isoform diversity

GSDMs share conserved pore-forming and regulatory domains with other gasdermin proteins. Isoform diversity critically influences function; for example, specific GSDMB isoforms containing exon 6 execute pyroptosis, whereas others lacking this exon promote tumor progression and are associated with poor outcomes [38, 59]. This structural heterogeneity underlies the contrasting roles of different GSDMs.

#### Subtype-specific functional roles

GSDMs contribute to the subtype-specific molecular landscape of cancers. GSDMC upregulation is associated with poor prognosis and immune infiltration in basal-like/TNBC [50]. GSDMD often promotes cell proliferation, whereas GSDME mediates chemotherapy-induced pyroptosis and antitumor immunity [60]. Functional analyses have revealed that GSDMs are involved in subtype-dependent pathways related to the immune response, metabolism, and drug resistance, with members such as GSDMA/B sometimes acting in opposition to GSDME [61].

## The role of pyroptosis in breast cancer progression

### The role of inflammasomes in the breast cancer tumor microenvironment

Inflammasomes are multiprotein complexes that act as crucial sensors of danger signals within the breast cancer tumor microenvironment (TME) [62]. Upon activation, they trigger the maturation of pro-inflammatory cytokines (IL-1β and IL-18) and induce pyroptosis [63]. This response plays a dual role in breast cancer progression, acting as a “double-edged sword” that either stimulates antitumor immunity or promotes tumorigenesis [64].

#### Inflammasome composition and activation in the breast cancer TME

Canonical inflammasomes consist of a sensor molecule (e.g., NLRP3), an adaptor protein ASC, and pro-caspase-1 [46]. In the breast cancer TME, these complexes are primarily activated by damage-associated molecular patterns (DAMPs) released from dying tumor cells, the remodeled extracellular matrix, or stressed cells under hypoxic conditions [65]. This activation triggers caspase-1, which cleaves pro-IL-1β and pro-IL-18 into their active forms, driving a potent inflammatory response [66]. The tight regulation of this pathway presents opportunities for therapeutic intervention.

#### NLRP3 in breast cancer

The NLRP3 inflammasome is a key sensor in breast cancer that is activated by TME stressors such as ATP, ROS, and crystals [67]. Its activation triggers caspase-1, leading to GSDMD cleavage and pyroptosis, accompanied by the release of IL-1β and IL-18, which remodels the TME [68]. Thioredoxin-interacting protein (TXNIP) is a significant upstream regulator of NLRP3, and targeting the TXNIP-NLRP3 axis represents a potential therapeutic strategy [69]. Beyond cancer cells, stromal fibroblasts expressing NLRP3 contribute to cancer progression by secreting pro-tumorigenic factors and recruiting immunosuppressive cells [65]. The complex crosstalk between NLRP3 and other pathways underscores the need to understand these interactions to develop effective pyroptosis-targeting therapies [70].

#### AIM2 and cytosolic DNA

The AIM2 inflammasome detects cytosolic double-stranded DNA, which can result from genomic instability or therapy-induced damage [71]. Its activation triggers caspase-1, leading to the maturation of IL-1β and IL-18 and the cleavage of GSDMD to initiate pyroptosis [68]. This inflammatory response can significantly remodel the tumor microenvironment, influencing cancer progression and antitumor immunity; however, its precise prognostic and therapeutic implications in breast cancer require further investigation [72].

#### Pyroptosis and its dual role in breast cancer

A key consequence of inflammasome activation is pyroptosis, a highly inflammatory form of cell death mediated by gasdermin D (GSDMD). Caspase-1 cleaves GSDMD, leading to pore formation, cell lysis, and the release of damage-associated molecular patterns (DAMPs) and pro-inflammatory cytokines, such as IL-1β and IL-18, into the tumor microenvironment [46]. The impact of pyroptosis on breast cancer is complex, exerting both antitumor and pro-tumor effects on breast cancer cells.

a. Antitumor Effects: Pyroptosis can stimulate anti-tumor immunity by releasing tumor-associated antigens (TAAs) and pro-inflammatory cytokines [3]. The released TAAs are taken up by antigen-presenting cells, such as dendritic cells, leading to the activation of tumor-targeting cytotoxic T lymphocytes [72]. Cytokines such as IL-1β and IL-18 promote the maturation and activation of immune cells, amplifying the immune response [55]. For instance, GSDMD-mediated pyroptosis increases IL-1β and IL-18 levels, correlating with increased immune cell infiltration and reduced immunosuppressive cells, such as MDSCs [73]. The release of damage-associated molecular patterns (DAMPs) during pyroptosis further enhances this effect. Extracellular ATP activates the P2X7 receptor on immune cells, promoting inflammation and recruitment of macrophages and dendritic cells [56]. Similarly, released HMGB1 can stimulate pattern recognition receptors on immune cells, bolstering anti-tumor immunity [74]. The combination of pyroptosis induction and immune checkpoint inhibitors is a promising strategy for enhancing T cell infiltration and tumor cell eradication in breast cancer.

b. Pro-tumor Effects: The release of pro-inflammatory mediators, such as IL-1β, can establish a chronic inflammatory microenvironment that promotes tumor progression [3]. This inflammation can induce DNA damage and genomic instability, thereby accelerating tumor evolution. Furthermore, IL-1β recruits monocytes that differentiate into tumor-associated macrophages, which support tumor growth and angiogenesis [75]. Chronic cytokine exposure activates pro-survival pathways, such as NF-κB and STAT3, thereby enhancing cancer cell proliferation and invasion [66]. Consequently, although pyroptosis can trigger antitumor immunity, the resultant inflammation may inadvertently fuel cancer progression.

#### Factors governing the pro- and anti-tumor switch of pyroptosis

Pryoptosis functions as both a tumor suppressor and promoter, with this duality determined by cellular, molecular, and microenvironmental factors. Understanding these determinants is essential for the development of effective therapeutic applications. Several factors influence whether pyroptosis promotes or suppresses tumorigenesis in a specific context.

a. *Nature and Duration of Inflammatory Response:* The type and persistence of the inflammatory response induced by pyroptosis are critical molecular and microenvironmental switches that dictate whether it exerts anti-tumor or pro-tumor effects. Acute pyroptotic activation, often initiated by inflammasomes, leading to rapid caspase-1 activation and GSDMD cleavage, generally leads to rapid cell death and the controlled, transient release of damage-associated molecular patterns and pro-inflammatory cytokines, such as IL-1β and IL-18. This acute burst stimulates robust antitumor immunity, thereby acting as a tumor suppressor [1]. This acute response can “warm” an immune-cold tumor, facilitating immune cell activation and recruitment [76]. For example, in preclinical studies, the induction of acute pyroptosis has been shown to suppress cancer development and progression [1]. The precise “timing, level, and composition of pyroptosis induction” are crucial determinants of this outcome [77].

Conversely, chronic or dysregulated pyroptosis, characterized by sustained inflammasome activation and prolonged, often excessive, release of inflammatory cytokines such as IL-1β, IL-18, and DAMPs such as HMGB1, contributes to a persistent inflammatory microenvironment [78]. This sustained inflammatory milieu acts as a significant microenvironmental switch, fostering tumorigenesis by promoting immune escape, tumor cell growth, invasion, and metastasis, and contributing to drug resistance [77]. Mechanistically, while both IL-1β and IL-18 are initially immune-activating, the prolonged and high-level presence of IL-1β can pivot the response towards pro-tumorigenesis by establishing a chronic pro-inflammatory and immunosuppressive environment, influencing immune cell differentiation (e.g., recruitment of myeloid-derived suppressor cells and tumor-associated macrophages) [66]. In contrast, IL-18 is largely associated with robust antitumor immune responses, and its sustained presence is generally beneficial [73]. This persistent inflammatory milieu, often driven by dysregulated inflammasomes and specific cytokine kinetics, supports the tumor microenvironment through the sustained production of inflammatory cytokines, which can promote breast cancer initiation and progression [77]. Clinical observations further underscore this, suggesting that chronic inflammation and persistent infections are closely associated with cancer onset, proliferation, aggression, and angiogenesis [79].

b. *Specific Inflammatory Mediators and Their Concentrations:* The specific balance and precise concentrations of pro-inflammatory cytokines released during pyroptosis, particularly IL-1β and IL-18, function as critical molecular switches that determine the ultimate pro- or anti-tumor outcomes [73]. Pyroptosis is primarily executed by gasdermin proteins, whose cleavage by specific caspases acts as a fundamental molecular switch that governs the release of inflammatory mediators [80]. For instance, canonical and non-canonical inflammasome activation leads to caspase-1/4/5/11 cleavage of GSDMD, whereas caspase-3 and caspase-8 can cleave GSDME, and caspase-8 can also cleave GSDMC, leading to distinct pyroptotic pathways [55]. This specific caspase-gasdermin axis dictates the mode of cell death and subsequent cytokine release.

While both IL-1β and IL-18 contribute to immune cell recruitment, excessive or prolonged release of IL-1β can mechanistically pivot pyroptosis towards a pro-tumorigenic role [77]. This pro-tumor effect of IL-1β is primarily mediated by its ability to foster a chronic pro-inflammatory and immunosuppressive tumor microenvironment, notably through the recruitment of myeloid cells, which can promote tumor growth and metastasis [3]. Preclinical models have consistently demonstrate IL-1β‘s role in driving tumor growth, invasion, and metastasis [77]. Conversely, IL-18 is largely recognized for its potent ability to promote robust antitumor immune responses by activating immune cells, making it a target for therapeutic strategies [66].

In addition to molecular mediators, microenvironmental factors also serve as critical switches. For example, hypoxia, a common feature of the tumor microenvironment, can act as a significant environmental switch. It can drive PD-L1-mediated apoptosis-to-pyroptosis conversion, which may paradoxically contribute to chronic tumor necrosis and promote tumor growth while impeding antitumor immunity [76]. This differential impact underscores the need for the “timing, level and composition of pyroptosis induction need to be closely controlled” [77], as these parameters influence the activation of specific caspase-gasdermin axes, the balance of cytokine release, and the contextual microenvironmental responses. Elevated serum IL-1β and IL-18 levels have been identified as biomarkers of post-irradiation pyroptosis in patients with breast cancer [81].

c. *Tumor Cell Intrinsic Factors and Genetic Landscape:* The inherent characteristics of tumor cells, including their genetic mutations, specific oncogenic pathways, and epigenetic modifications, represent crucial intrinsic “switches” that dictate the outcome of this process. Some cancers actively downregulate or express non-functional forms of gasdermin proteins, which are essential executioners of pyroptosis, as a sophisticated mechanism of immune evasion and survival [76]. Mechanistically, gasdermins such as GSDMD, GSDMB, and GSDME function as pore-forming effector proteins. Upon proteolytic cleavage by caspases or granzymes, their N-terminal domains oligomerize to form pores in the cell membrane, leading to cell lysis and the release of pro-inflammatory contents [56].

**Fig. 3:**
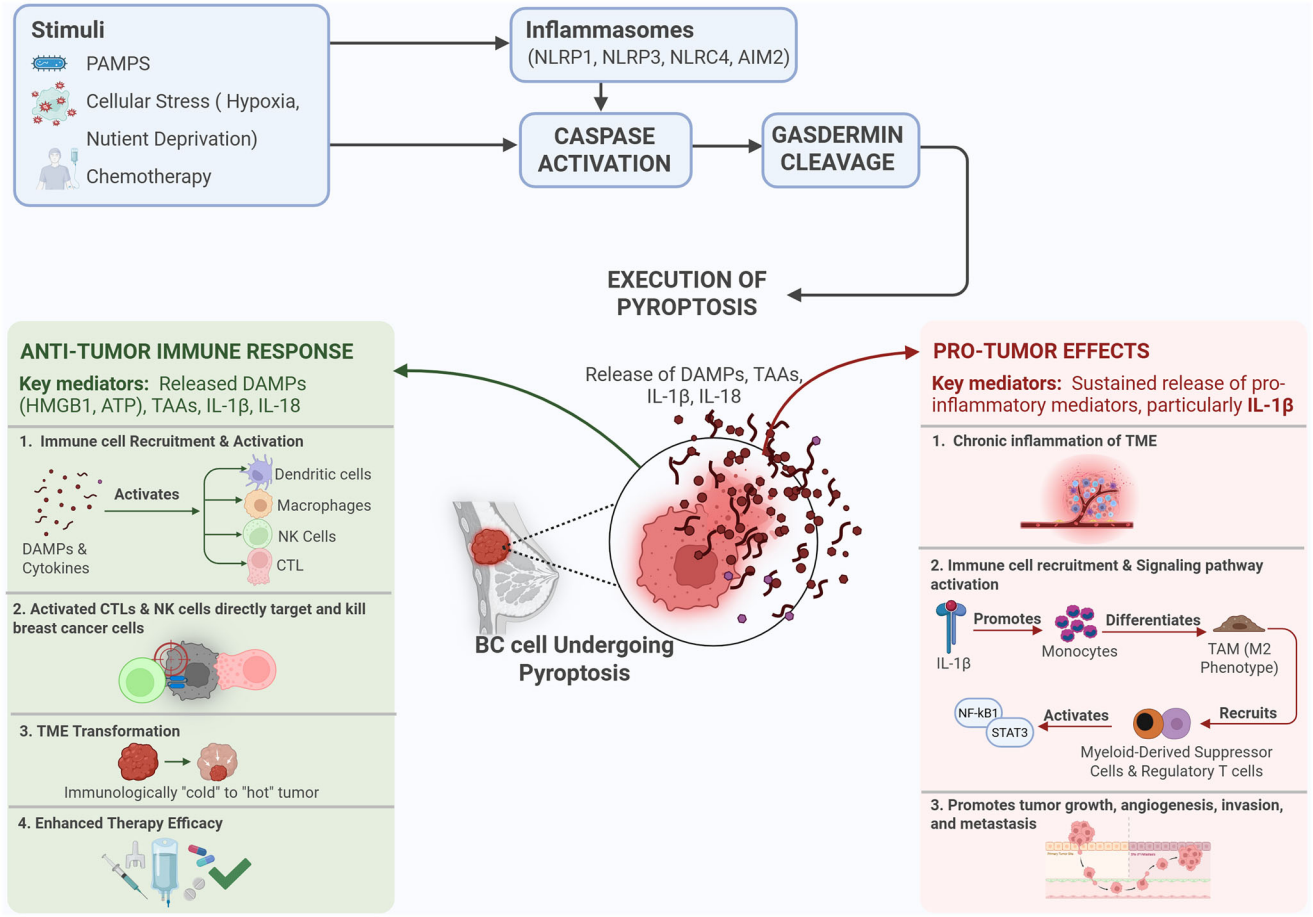
Immunity associated with pyroptosis in breast cancer. Cellular stressors, such as chemotherapy, hypoxia, and nutrient deprivation, activate inflammasomes (NLRP1, NLRP3, NLRC4, and AIM2), triggering caspase activation and gasdermin cleavage. Pyroptosis releases DAMPs (HMGB1 and ATP), tumor-associated antigens (TAAs), and cytokines (IL-1β and IL-18), which initiate divergent immune outcomes. DAMPs and cytokines recruit and activate dendritic cells, macrophages, NK cells, and cytotoxic T lymphocytes (CTLs), enhancing antitumor immunity, transforming immunologically “cold” tumors to “hot,” tumors, and improving therapy responsiveness. Sustained IL-1β release promotes chronic inflammation, recruits M2-type macrophages and myeloid-derived suppressor cells, activates NF-κB/STAT3 signaling, and facilitates tumor progression, angiogenesis, and metastasis. The figure summarizes the opposing immune responses elicited by pyroptosis in breast cancer, highlighting the need for balanced therapeutic strategies.

Genetic variations and epigenetic abnormalities, such as single nucleotide variations, copy number variations, and DNA methylation levels in pyroptosis-related genes, significantly influence prognosis and dictate the tumor response to pyroptosis induction [42]. For instance, differential expression patterns of gasdermin family members have been observed across different cancer subtypes. In breast cancer, GSDMD is In flammasome-mediated cytokine releaseoften upregulated, whereas GSDMB and GSDME are frequently downregulated in tumor samples compared to normal tissues [82]. GSDMB overexpression is associated with poor clinical outcomes in patients with HER2-positive breast cancer [68]. This differential expression can function as a molecular switch. For example, the presence of GSDME is critical because it can convert caspase-3-mediated apoptosis to pyroptosis in cancer cells, thereby activating antitumor immunity [83]. Conversely, GSDME deficiency has been associated with larger tumors [46]. Furthermore, epigenetic modifications, such as GSDME methylation, can serve as biomarkers for tumor detection, diagnosis, and prognosis, and reversing its silencing through demethylating agents can restore pyroptotic sensitivity [83]. Different gasdermin family members can also divergently influence therapeutic responses, with GSDMA and GSDMB regulating drug resistance in the opposite direction to that of GSDME [84]. Therefore, the tissue and genetic backgrounds of tumors profoundly alter the effects and therapeutic potential of pyroptosis.

d. *Tumor Microenvironment Context:* The cellular and molecular composition of the tumor microenvironment is a crucial determinant, acting as a complex array of “switches” that profoundly influences the outcome of pyroptosis. The delicate balance between pro-tumorigenic and anti-tumor factors within the TME dictates tumor growth and progression [56]. Pyroptosis can either “transform the tumor immune microenvironment from a “cold” to a “hot” state” [76], enhancing antitumor immunity by promoting immune cell infiltration and activation, or create an environment conducive to tumor growth and resistance (Fig. 3) [78].

A key molecular switch lies in the balance and sustained release of these inflammatory cytokines. Although IL-1β and IL-18 are both released during pyroptosis, their precise concentrations and timing critically influence the TME [73]. Excessive or prolonged release of IL-1β can mechanistically pivot pyroptosis towards a pro-tumorigenic role by fostering chronic inflammation and enhancing the recruitment of immunosuppressive cells, such as myeloid-derived suppressor cells. In contrast, IL-18 is recognized for promoting robust antitumor immune responses by activating immune cells [66].

The temporal switch of pyroptosis, which distinguishes between acute and chronic induction, also dictates its impact. Acute pyroptosis induction is generally associated with tumor suppression by evoking strong antitumor immunity and facilitating immune cell recruitment, converting a ‘cold’ tumor into a ‘hot’ tumor [82]. Preclinical studies have demonstrated that pyroptosis in a small percentage of tumor cells can lead to the complete eradication of breast graft tumors [82]. Conversely, chronic pyroptosis establishes a persistent inflammatory microenvironment that promotes tumorigenesis, immune escape, and resistance to therapy [77]. This chronic inflammation, often orchestrated by inflammasomes, can subvert immune responses and provide an advantage to tumor cells.

Furthermore, cellular and contextual switches within the TME play significant roles. The recruited MDSCs accumulate in the TME, where they potently inhibit the anti-cancer functions of T cells and natural killer cells, thereby promoting tumor growth, immune evasion, and resistance to therapy [85]. Specific microenvironmental conditions, such as hypoxia, can also act as a switch, driving PD-L1-mediated apoptosis-to-pyroptosis conversion. This switch can lead to chronic tumor necrosis, which paradoxically promotes tumor growth and impedes antitumor immunity [76].

e. *Caspase Activity and Gasdermin Cleavage:* Specific caspase activation and subsequent cleavage of gasdermin family proteins are central to the execution of pyroptosis. Upon stimulation, inflammatory caspases, specifically caspase-1, caspase-4, caspase-5, and caspase-11, primarily cleave GSDMD to initiate pyroptosis [3]. The activation of caspase-1 is typically mediated by inflammasomes (canonical pathway), while caspase-4, -5, and -11 can be directly activated by lipopolysaccharide via a non-canonical pathway [76]. This proteolytic cleavage liberates the N-terminal pore-forming domain of GSDMD, which oligomerizes to form pores in the cell membrane [33]. These pores, approximately 21 nm in diameter, lead to cell lysis and the release of pro-inflammatory contents such as IL-1β, IL-18, HMGB1, and ATP [86].

In contrast, GSDME is typically cleaved by apoptotic caspase-3, which possesses the unique ability to convert caspase-3-mediated apoptosis into pyroptosis in cancer cells, thereby activating antitumor immunity [83]. The cellular level of GSDME acts as a critical molecular switch: high GSDME levels drive pyroptosis following caspase-3 activation, whereas low levels favor apoptosis [76]. Caspase-8 exhibits broader specificity and can cleave both GSDMD and GSDME [76]. It is a key hub in the complex network involving apoptosis, necroptosis, and pyroptosis [87]. Additionally, granzyme B, released by natural killer cells and cytotoxic T lymphocytes, can activate caspase-3 to cleave or directly cleave GSDME, leading to pyroptosis [66]. Granzyme A can cleave GSDMB and GSDMC, respectively [56], and caspase-8 can also cleave GSDMC [55]. Other proteases, such as neutrophil elastase and cathepsin G, can also cleave GSDMD [71].

The intramolecular interaction between the N-terminal- and C-terminal fragments of gasdermins normally prevents the activation of their pore-forming activity. Upon proteolytic cleavage by specific caspases or granzymes, the cytotoxic N-terminal domain is liberated and oligomerizes in the cell membrane, forming large pores [76]. This precise activation, alongside variations in these activation pathways, the expression of different gasdermins, and context-dependent cleavage specificity, represents a critical molecular switch leading to diverse cellular effects and influencing the overall pro- or anti-tumor outcome [3]. For example, the precise mechanisms by which inflammatory caspases trigger pyroptosis have been clarified by the discovery of GSDMD as a requirement for caspase-1-mediated pyroptosis [88]. Furthermore, the presence of specific gasdermins, such as GSDMA3, even in a small percentage of tumor cells, can trigger potent antitumor immunity [71], highlighting the importance of the specific gasdermin type in determining the therapeutic response.

Further research into these intricate regulatory mechanisms and “switches” is essential for the precise manipulation of pyroptosis for therapeutic benefits in cancer treatment.

#### Inflammasome-mediated cytokine release and immune modulation in breast cancer

The release of IL-1β and IL-18 by activated inflammasomes significantly affects the TME of breast cancer [63]. IL-1β promotes angiogenesis, stimulates the production of matrix metalloproteinases, and enhances the recruitment of myeloid-derived suppressor cells [3]. These effects contribute to tumor growth, invasion, and metastasis. IL-18 signals through MyD88 and NF-κB, enhancing NK cell activity and promoting Th1 responses, potentially leading to antitumor immunity [73]. However, it is crucial to note that IL-18 signaling without concomitant inflammatory cytokines may enhance Th2 responses. The balance between these pro- and anti-tumorigenic effects depends on the specific context and interplay with other factors in the TME [89]. Complex interactions within the TME influence the mechanisms of immune evasion and the response to immunotherapy.

### Pyroptosis and metastasis

Reflecting its established dual role, pyroptosis plays a complex and context-dependent role in cancer metastasis, acting as a “double-edged sword” [1]. The precise mechanisms governing its influence on metastasis are multifaceted and involve a delicate balance between pro- and antitumorigenic effects [73]. A deeper understanding of these mechanisms is crucial for developing effective therapeutic strategies.

#### Mechanisms of pyroptosis in metastasis

a. Prometastatic effects: Chronic pyroptosis can contribute to a pro-tumorigenic microenvironment by releasing inflammatory cytokines, such as IL-1β, IL-18, and HMGB1 [1]. This inflammatory environment promotes tumor growth, angiogenesis, invasion and metastasis. These cytokines activate signaling pathways that enhance cancer cell survival, proliferation, and migration [90]. For example, HMGB1 released during pyroptosis has been shown to promote tumorigenesis, and in breast cancer, HMGB1 and Toll-like receptor 4 signaling are critically involved in the metastatic potential [91]. Furthermore, chronic inflammation resulting from persistent pyroptosis can lead to genomic instability and epigenetic changes, promoting metastasis [77]. Recent clinical data underscore the clinical relevance of this phenomenon; for instance, elevated levels of GSDMD-N in serum samples from patients with TNBC correlate with reduced overall survival and increased distant metastasis [81].

b. Anti-metastatic effects: In contrast, the induction of acute pyroptosis suppresses cancer development and metastasis [1]. Several anticancer drugs, including cisplatin, trigger pyroptosis, thereby restricting the growth and spread of malignant cells [92]. The acute release of intracellular contents during pyroptosis elicits a strong antitumor immune response, leading to cancer cell clearance [34]. This is often associated with the release of DAMPs, which activate immune cells and promote tumor cell recognition and elimination. For example, pyroptosis-mediated release of IL-1β stimulates dendritic cell maturation, which is crucial for antigen presentation and T cell activation [55]. Activated DCs present tumor-associated antigens to T cells, initiating a robust cytotoxic T lymphocyte response that targets and eliminates metastatic cells [93]. This process, known as cross-priming, can result in a systemic antitumor immune response [94]. Moreover, pyroptosis-induced ATP release activates the P2X7 receptor on immune cells, leading to NLRP3 inflammasome activation and amplification of the inflammatory response, thereby enhancing immune cell accumulation in tumors [95].

**Fig. 4:**
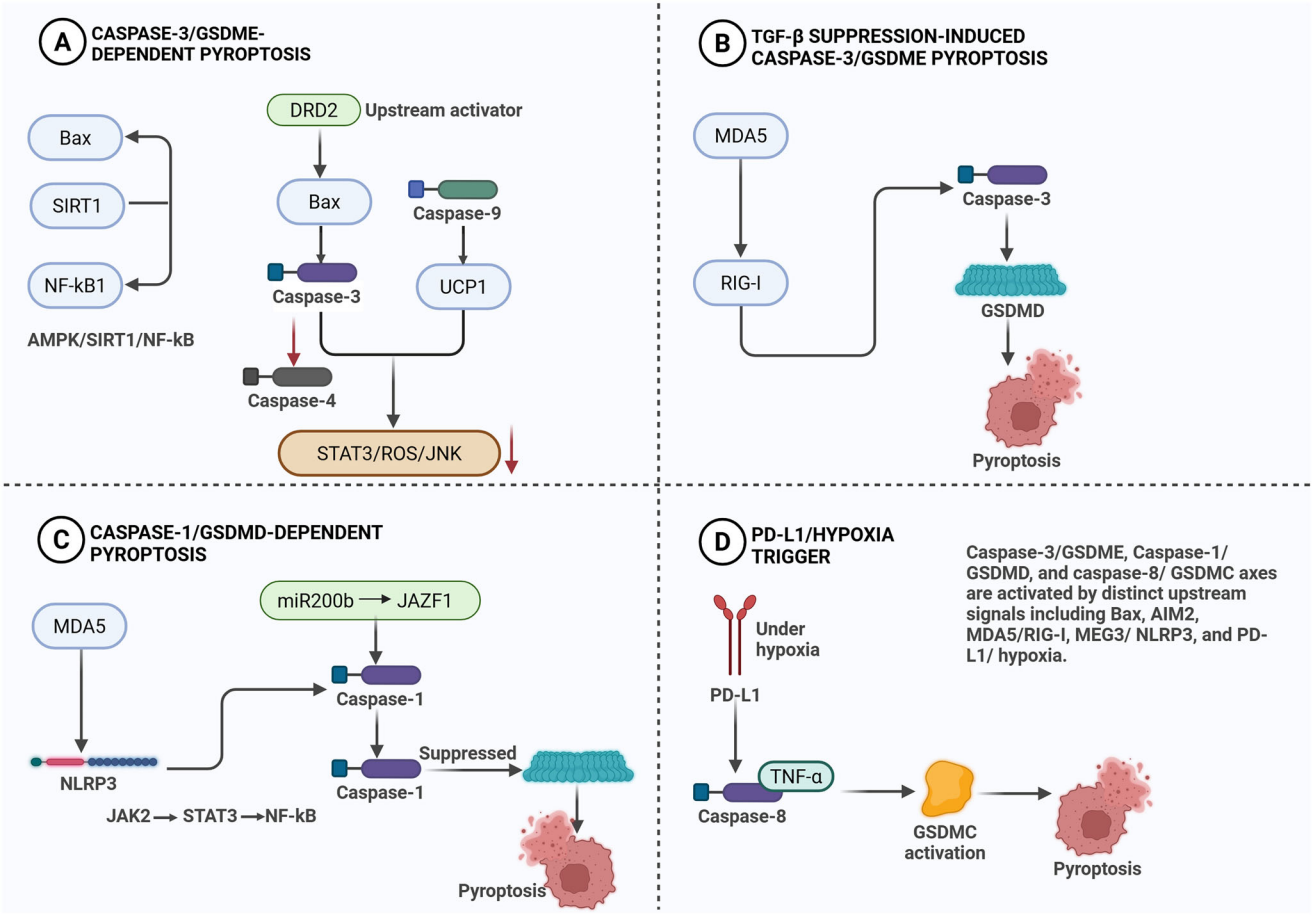
Signaling pathways that regulate pyroptosis. **A** Upstream signals, including Bax, AIM2, DRD2, and UCP1, along with the AMPK/SIRT1/NF-kappaB and STAT3/ROS/JNK signaling pathways, contribute to the induction of caspase-3/GSDME-dependent pyroptosis. **B** Inhibition of the TGF-β signaling pathway activates MDA5 and RIG-I, triggering GSDME-mediated pyroptosis by inducing caspase-3. **C** Concurrently, the MEG3/NLRP3, miR-200b/JAZF1, and JAK2/STAT3/NF-kappaB pathways regulate caspase-1/GSDMD-dependent pyroptosis. **D** Furthermore, hypoxia-induced alterations in PD-L1 facilitate the conversion of TNF-α-induced apoptosis to pyroptosis by promoting caspase-8 activation and the proteolytic cleavage of GSDMC.

Clinical trials have begun to explore the potential of pyroptosis-inducing agents in the treatment of metastatic breast cancer. Although these trials are still in their early stages, preliminary results suggest that combination therapies, including pyroptosis-inducing agents and immune checkpoint inhibitors, may be particularly effective in patients with advanced disease [92]. For instance, a Phase I clinical trial evaluating cisplatin and pembrolizumab in patients with metastatic TNBC showed promising results, with a subset of patients experiencing durable responses and significant tumor burden reduction [96]. Further research is needed to fully understand the role of pyroptosis in the anti-metastatic response and to identify predictive biomarkers. As shown in Fig. 4, the regulation of pyroptosis in breast cancer involves multiple signaling axes that converge on gasdermin activation. Canonical inflammasome pathways (e.g., NLRP3–caspase-1–GSDMD) and non-canonical routes (e.g., caspase-4/5/11) trigger inflammatory pyroptosis, whereas apoptotic caspase-3 and immune cell-derived granzymes activate GSDME or GSDMB to convert The diagram also highlights the regulatory networks involving ROS generation, NF-κB and STAT3 signaling, and the crosstalk between tumor microenvironment stressors, such as hypoxia, PD-L1 translocation, and cytokine feedback. Together, these pathways determine whether pyroptosis acts as a tumor-suppressive or pro-inflammatory event in breast cancer progression.

## Strategiess for targeting pyroptosis in breast cancer therapy

Current treatments targeting pyroptosis in breast cancer involve the activation or blocking of pyroptosis to fight tumors or reduce unwanted effects [1, 37]. A growing number of studies have described new mechanisms of programmed cell death, including pyroptosis, suggesting a promising approach for future cancer treatment. Treatments can be broadly classified based on their mechanisms of action and include chemotherapy drugs, small-molecule inhibitors, immunotherapies, and nanomedicine approaches. Each approach aims to leverage the unique aspects of the pyroptosis pathway to maximize therapeutic efficacy and minimize off-target effects [52]. Table 3 lists representative chemotherapeutics, nanodrugs, and photosensitizers capable of modulating pyroptosis. Including their principal mechanisms beyond gasdermin activation helps delineate the crosstalk between pyroptosis and other death pathways.

**Table 3:** Therapeutic Agents Modulating Pyroptosis for Breast Cancer Treatment.

Therapeutic Agent	Pyroptosis Pathway	Main Mechanism of Action (beyond pyroptosis induction)	Breast Cancer Subtype	Clinical Trial Phase	Breast Cancer-Specific Evidence	Ref
Disulfiram–Cu2 + (IC@PCH NPs)	NLRP3/caspase-1 pathway	Primarily induces cuproptosis via copper accumulation leading to proteotoxic stress and mitochondrial dysfunction; can also induce ferroptosis	TNBC	Preclinical	Significant antitumor effect via pyroptosis in TNBC models	[138, 221]
Cytarabine + Chlorin e6 (A-C/NPs)	GSDME-mediated, ROS-induced	Chemotherapeutic agent; Photosensitizer for PDT	Breast cancer (mouse model)	Preclinical	Suppressed orthotopic, abscopal, and recurrent tumors	[222]
Mitoxantrone + Gambogic acid (nanococrystals)	Ribosomal stress, pyroptosis gene regulation	Chemotherapeutic agent; Natural compound	TNBC (models)	Preclinical	Triggered pyroptosis cascade immune effects in TNBC	[223]
Dihydroartemisinin (DHA)	AIM2/caspase-3/GSDME axis	Antimalarial agent with anticancer properties	MCF-7, MDA-MB-231, xenograft mice	Preclinical	Inhibited proliferation, induced pyroptosis in vitro/in vivo	[224]
Cisplatin (DDP)	MEG3/NLRP3/caspase-1/GSDMD pathway	Primarily forms DNA cross-links and adducts, leading to DNA damage and inducing apoptosis	TNBC (in vitro, in vivo)	Preclinical	Induced pyroptosis, suppressed tumor growth/metastasis	[51]
IR780-ZnS@HSA (albumin nanoparticles)	Caspase-3–GSDME, cGAS–STING pathway	Photosensitizer for Photothermal Therapy and/or Photodynamic Therapy via near-infrared light absorption	TNBC (4T1 mouse model)	Preclinical	Inhibited tumor growth, improved aPD-L1 efficacy	[120]
Doxorubicin + Decitabine (FPSD NPs)	GSDME upregulation, pyroptosis induction	Chemotherapeutic agent; DNA methyltransferase inhibitor	4T1 breast cancer (mouse model)	Preclinical	Reduced tumor volume, promoted antitumor immunity	[53]
HfO2 NPs + Decitabine	Caspase-3 activation, GSDME demethylation	Nanoparticle-based agent (HfO2 NPs); DNA methyltransferase inhibitor	TNBC (models)	Preclinical	Converted apoptosis to pyroptosis, inhibited metastasis	[157]
T-P (prodrug photosensitizer)	ROS-induced pyroptosis in CSCs	Photosensitizer for Photodynamic Therapy generating reactive oxygen species upon light activation	Breast cancer stem cells (in vivo)	Preclinical	Eliminated primary tumor, inhibited distant growth	[225]
R@L-MRS17 (self-adaptor nanoplatform)	ROS-induced pyroptosis, macrophage reeducation	Involves Photodynamic Therapy due to reactive oxygen species generation	Breast cancer (bilateral tumors)	Preclinical	Suppressed tumor growth/metastasis, enhanced ICB	[226]

a. Chemotherapy-Induced Pyroptosis

Many conventional chemotherapeutic drugs induce cancer cell death through various mechanisms, and increasing evidence suggests that pyroptosis significantly contributes to their efficacy in breast cancer therapy [52]. Anthracyclines, platinum-based drugs, and taxanes trigger pyroptosis by activating the inflammatory signaling pathways. For instance, doxorubicin induces DNA damage and oxidative stress, leading to the activation of the NLRP3 inflammasome and GSDMD cleavage [35]. Cisplatin forms DNA adducts that activate the AIM2 inflammasome, resulting in gasdermin D (GSDMD)-mediated pyroptosis [97]. Taxanes, such as paclitaxel, disrupt microtubule dynamics, leading to mitotic arrest and caspase-3 activation, which cleaves and triggers pyroptosis in cells expressing GSDME [98].

Recent studies have focused on optimizing chemotherapy regimens to enhance pyroptosis induction. Combination therapies, including chemotherapeutic drugs and agents that sensitize cancer cells to pyroptosis, have shown promise. Chemotherapeutic drugs can induce pyroptosis via the caspase-3/GSDME pathway in cells with high GSDME expression [37], highlighting the distinct mechanisms compared to inflammasome-mediated GSDMD cleavage. While cisplatin primarily activates GSDMD-mediated pyroptosis via inflammasomes, it can also induce GSDME-mediated pyroptosis through caspase-3 in specific cellular contexts or at specific concentrations [98]. Moreover, epigenetic modulation strategies, such as the use of DNA methyltransferase inhibitors to upregulate GSDME expression, can restore pyroptotic sensitivity in cancer cells in which this gene has been silenced [66]. Decitabine promotes DNA hypomethylation to upregulate GSDME, and when combined with cisplatin in nanoliposomes, it increases pyroptosis and cytotoxic T lymphocyte infiltration in TNBC models [66]. The precise mechanisms are complex and depend on the specific drug and the tumor microenvironment [99]. For instance, doxorubicin facilitates intracellular ROS accumulation, leading to JNK phosphorylation, caspase-3 activation, and GSDME cleavage [99].

In TNBC, intrinsic resistance to conventional therapies makes pyroptosis induction an attractive therapeutic strategy [100]. Cisplatin induces pyroptosis via the MEG3/NLRP3/caspase-1/GSDMD pathway, thereby suppressing tumor growth and metastasis [51]. Furthermore, HDAC inhibitors [101], tetraarsenic hexoxide [102], and novel compounds such as nigericin [103] have been shown to induce pyroptosis in TNBC, inhibit tumor growth, and enhance antitumor immune responses. These findings underscore the potential of manipulating pyroptotic pathways to improve the efficacy of chemotherapy regimens in TNBC.

b. Small Molecule Inhibitors Targeting Pyroptosis

Small-molecule inhibitors targeting specific components of the pyroptosis pathway offer a refined approach for modulating pyroptosis in breast cancer. These inhibitors can fine-tune the pyroptotic process by either dampening excessive inflammation or amplifying pyroptosis in cancer cells, thereby facilitating tumor cell death [34].

Inhibition of the NLRP3 inflammasome has garnered significant attention because of its pivotal role in activating caspase-1 and initiating pyroptosis [104]. MCC950, a highly selective NLRP3 inhibitor, has shown promise in preclinical studies for attenuating inflammation and impeding cancer progression [105]. Other compounds, such as glyburide and Bay 11-7082 also exhibit inhibitory effects on NLRP3 inflammasome assembly and activation [105].

Directly targeting caspase-1 is another approach to modulating pyroptosis. VX-765, a caspase-1 inhibitor evaluated in clinical trials for inflammatory conditions, holds potential for mitigating the pro-tumorigenic consequences of pyroptosis [106]. Furthermore, GSDMD inhibitors, such as disulfiram, can impede pyroptosis by obstructing GSDMD pore formation, thereby preventing the release of inflammatory intracellular content [104].

The development of highly selective and effective small-molecule inhibitors is a key focus of precision cancer therapies. Fine-tuning pyroptosis to achieve the desired therapeutic outcome may be achieved through careful selection of targets and the design of inhibitors with optimal pharmacokinetic and pharmacodynamic properties [88].

c. Immunotherapy Combined with Pyroptosis-Inducing Agents

Combining immunotherapy with pyroptosis-inducing agents is a compelling strategy for enhancing anti-tumor immunity and improving therapeutic outcomes in breast cancer patients. Immune checkpoint inhibitors (ICIs) aim to unleash the immune system by disrupting inhibitory signals; however, their efficacy in breast cancer is often limited by an immunosuppressive tumor microenvironment (TME) [107]. Pyroptosis counteracts this by releasing tumor-associated antigens and pro-inflammatory cytokines, effectively converting immunologically “cold” tumors into “hot” tumors, thereby enhancing the efficacy of immunotherapy [56].

This synergy is particularly relevant for TNBC, which often presents with a “cold” TME [108]. The pyroptosis-mediated release of cytokines, such as IL-1β and IL-18, can transform the immunosuppressive microenvironment of TNBC into an immune-responsive state [1]. For instance, inducing pyroptosis in TNBC cells significantly improves the efficacy of anti-PD-L1 therapy by increasing T cell infiltration [106]. Similarly, agents such as nigericin [107] and HDAC inhibitors [106] have been shown to induce pyroptosis and stimulate synergistic antitumor immune responses when combined with ICIs in TNBC models.

In addition to small molecules, other modalities can induce immunogenicity. Chemotherapeutic agents can trigger pyroptosis, leading to immune cell activation, providing a rationale for their combination with ICIs [56]. Oncolytic viruses (OVs), which selectively infect and kill cancer cells, can also induce pyroptosis, and their combination with ICIs has demonstrated synergistic effects in preclinical models [82]. Furthermore, adoptive cell therapies, such as those involving CAR-T cells, can be combined with pyroptosis-inducing agents, as the pyroptotic generation of neoantigens can augment the formation of antigen-specific cytotoxic T lymphocytes, thereby enhancing CAR-T cell efficacy [56].

However, it is imperative to precisely regulate pyroptosis to prevent excessive inflammation and potential off-target effects of the treatment. Future studies should focus on identifying predictive biomarkers and elucidating the molecular mechanisms of pyroptosis to develop more effective and targeted combination therapies.

d. Nanotechnology-Based Drug Delivery Systems for Targeted Pyroptosis Induction

Nanotechnology-based drug delivery systems enable targeted pyroptosis induction in breast cancer, delivering therapeutic agents to tumors while reducing off-target effects of the treatment. Nanoparticles encapsulate drugs and enhance tumor accumulation through targeting [109]. These systems, with biomimetic coatings and targeting ligands, allow precise control of pyroptosis [110], focusing on cancer cells to enhance tumor killing and antitumor immunity.

i. Biomimetic Nanoparticles for Targeted Pyroptosis

Biomimetic nanoparticles, such as those coated with cancer cell membranes or other biological components, leverage natural biological recognition for improved tumor targeting and reduced immunogenicity, focusing on pyroptotic activity and subsequent immune activation at the tumor site [111]. Recent advancements include the development of tumor-membrane-targeted photosensitive dimers. These systems can precisely deliver pyroptosis-inducing agents to cancer cells, enhancing immunogenic cell death upon activation by external stimuli. A notable example is a tumor-membrane-targeted photosensitive dimer that enables pyroptosis-mediated synergistic photodynamic and photothermal immunotherapies [112]. This approach strategically targets cancer cell membranes, allowing for the localized induction of pyroptosis upon activation, thereby converting immunologically “cold” tumors into “hot” tumors.

ii. Stimuli-Responsive Nanoplatforms

Stimuli-responsive nanoplatforms afford spatiotemporal precision in pyroptosis induction by coupling tumor microenvironment cues or exogenous triggers to material reconfiguration, thereby gating inflammasome signaling cascades while curtailing systemic inflammation [113, 114]. Endogenous stimuli, such as acidic pH, elevated glutathione levels, matrix metalloproteinases, or reactive oxygen species (ROS), provoke supramolecular disassembly, charge inversion, or self-assembly, culminating in endolysosomal perturbation and canonical or non-canonical pyroptosis [55, 115, 116].

For instance, MMP-2/GSH tandem-responsive nanoparticles first undergo extracellular proteolytic cleavage, followed by intracellular reductive disulfide scission to release payloads. This process drives a negative-to-positive ζ-potential reversal and nanoparticle-to-non-peptide nanofiber transformation within lysosomes [114]. The resulting rigid nanofibers impale lysosomal membranes, causing cathepsin B leakage, NLRP3/ASC/caspase-1 inflammasome priming, GSDMD-NT oligomerization, plasma membrane poration, IL-1β/IL-18 maturation, osmotic lysis, and immunogenic cell death [114].

Similarly, H⁺/GSH dual-responsive DNAzyme nanocomplexes are dismantled in acidic/reductive endosomes, liberating Zn²⁺/Mn²⁺ cofactors, which enable ATG5 mRNA cleavage by cascade DNAzymes, thereby blocking autophagic flux [115]. Concurrently, O₂ generation alleviates hypoxia, whereas TMPyP4-mediated ¹O₂ surges trigger NLRP3 inflammasome nucleation and caspase-1/GSDMD activation [115].

Acid-activatable nanophotosensitizers exemplify pH-gating: lysosomal acidification hyperactivates endosomal phospholipase C, promoting photosensitizer egress to early endosomes. Subsequent light-triggered ROS then induce GSDME cleavage prior to lysosomal fusion, thereby averting non-specific late-endosomal pyroptosis [116].

Exogenous modalities further amplify this paradigm of treatment. For example, ROS-responsive nanosystems [117] and aggregation-induced emission luminogen-based photoactivatable theranostics [118] harness near-infrared irradiation for selective ¹O₂ amplification. This leads to Tom20 oxidation or NLRP3 priming, followed by caspase-3/GSDME or caspase-1/GSDMD activation, respectively, and promotes cGAS-STING crosstalk for type I IFN potentiation [55, 119]. These transformations bypass efflux-mediated resistance, amplify DAMPs, and synergize with checkpoint blockade to yield abscopal effects in TNBC models [55, 66]. Ligand functionalization further refines homing, effectively merging pyroptosis and adaptive immunity.

iii. Self-Assembly and Aggregation-Based Nanoplatforms Compared to Conventional Carrier-Based Nanoparticles

Self-assembly and aggregation-based nanoplatforms represent an innovative paradigm shift from conventional carrier-based nanoparticles (e.g., liposomes, polymeric micelles such as PLGA cores [111], or stimuli-responsive systems [117]) by harnessing supramolecular chemistry to directly impose mechanical stress on cellular membranes, thereby activating inflammasome pathways without relying primarily on encapsulated drug payloads [113, 114]. Triggered by tumor-specific cues such as pH, enzymes, or reducing agents, these platforms undergo in situ supramolecular assembly into rigid nanostructures such as nanofibers, disrupting membrane fluidity and integrity to stimulate NLRP3 inflammasome oligomerization, caspase-1 activation, and GSDMD cleavage, culminating in pore formation, osmotic lysis, and immunogenic release of danger signals [114]. For example, tumor-membrane-targeted photosensitive dimers enable pyroptosis-synergized photodynamic/photothermal effects [112, 114], offering spatiotemporal precision in heterogeneous breast cancer microenvironments.

Critically, while conventional carrier-based nanoparticles predominantly induce pyroptosis indirectly via the controlled release of chemotherapeutic agents (e.g., cisplatin-decitabine liposomes upregulating GSDME [111]) or photosensitizers generating ROS/caspase-3 activation [120], self-assembling systems provide a more direct physical mechanism that bypasses drug resistance pathways associated with metabolic adaptations in TNBC [37, 113].

Self-assembly platforms have demonstrated heightened potency in preclinical TNBC models by eliciting stronger antitumor immunity through robust DAMP/cytokine release [114, 120]. However, their controllability lags behind that of mature carriers due to unpredictable aggregation in vivo, potentially exacerbating cytokine storms [113, 121]. In contrast, conventional systems offer superior safety profiles with FDA-approved precedents but suffer diminished potency against resistant tumors reliant on efflux pumps [37]. Hybrid designs integrating both approaches may optimize outcomes, although clinical translation requires rigorous biodistribution studies [122].

Biomimetic nanoparticles demonstrate superior targeting efficiency through homologous tumor membrane coatings, enabling precise homing and low immunogenicity, while potently modulating immunity via robust DAMP release and T-cell infiltration in breast cancer models [55, 111]. Stimuli-responsive nanoplatforms excel in controllability, leveraging TME cues such as MMP-2/GSH or pH for spatiotemporal pyroptosis gating, balancing efficacy and safety by curtailing off-target inflammation [113, 114, 116]. In contrast, self-assembly/aggregation systems offer direct mechanical potency against drug-resistant TNBC via lysosomal nanofiber impalement and inflammasome hyperactivation, enhancing immune responses but trading safety for risks of unpredictable in vivo aggregation and cytokine storms [114, 120, 121].

Among biomimetic, stimuli-responsive, and self-assembly based strategies, stimuli-responsive nanoplatforms are the most promising for the clinical translation of pyroptosis-based breast cancer therapy. These platforms achieve precise spatiotemporal control by responding to tumor-enriched cues, gating pyroptosis to minimize systemic inflammation, and maximizing inflammasome activation and DAMP release selectively in TNBC [113, 114, 116]. Unlike biomimetics, which rely on homologous targeting but risk incomplete specificity due to membrane heterogeneity, or self-assembly, which induces potent mechanical lysis but risks overactivation and cytokine storms [113, 121], stimuli-responsive systems balance potency and safety through predictable reconfiguration [115].

Stimuli-responsive designs leverage FDA-approved materials, facilitating scalability, reproducibility, and regulatory approval, compared to biomimetic manufacturing variability or self-assembly biodistribution unpredictability [123–125]. Preclinical data show robust TNBC efficacy with low toxicity, positioning them closest to the clinic amid nanomedicine’s <1% translation rate [126, 127].

In summary, the integration of biomimetic, stimuli-responsive, and self-assembling designs into nanoplatforms represents a transformative strategy for the precise induction of pyroptosis in breast cancer, enabling selective tumor cell elimination and robust antitumor immunity while minimizing off-target effects. However, despite significant preclinical promise, the clinical translation of these advanced nanomedicine systems faces several formidable challenges [126], particularly the limitations and risks inherent to pyroptosis induction. A primary concern is off-target inflammation, stemming from unintended pyroptosis in normal cells due to widespread gasdermin expression, especially in the gastrointestinal and hematopoietic tissues, which can precipitate tissue damage and chronic inflammatory states [66, 113]. The risk of cytokine storm is amplified by excessive inflammasome hyperactivation, leading to the hyper-release of pro-inflammatory mediators such as IL-1β and IL-18, potentially causing systemic toxicity and immune overactivation [73, 113, 121]. Nanoparticle accumulation poses another hurdle, as non-degradable inorganic components (e.g., metal-based nanomaterials) exhibit poor clearance, resulting in long-term organ toxicity, particularly hepatic sequestration, which undermines tumor-specific delivery [66, 122]. Moreover, unpredictable immune responses arise from biodistribution inconsistencies and irregular pyroptosis pathway expression across heterogeneous tumors, complicating their efficacy and safety profiles [73, 121]. Although engineering strategies, such as stimuli responsiveness, mitigate some risks [116], long-term biodistribution, toxicity monitoring, and precise targeting remain critical gaps that require advanced preclinical models that better recapitulate clinical heterogeneity [123]. This complexity also extends to manufacturing and scalability, where issues such as uncontrollable scaling, high production costs, and difficulties in ensuring batch-to-batch reproducibility hinder the transition from laboratory to large-scale clinical production [124]. Regulatory hurdles and the absence of standardized frameworks further impede progress, as the rapid evolution of nanotechnology often outpaces the development of clear regulatory guidelines and international standards [125]. Ultimately, the gap between successful preclinical outcomes and limited clinical authorizations highlights an insufficient understanding of nanomedicine-tumor interactions and calls for more robust preclinical models that better reflect clinical reality [123]. Addressing these challenges is crucial for realizing the full therapeutic potential of nanotechnology-based strategies for pyroptotic induction in breast cancer therapy.

e. Oncolytic Viruses for Pyroptosis Induction

Oncolytic viruses (OVs) are a class of self-replicating agents that selectively infect and lyse cancer cells, stimulating an antitumor immune response through the release of tumor-associated antigens [66]. The ability of certain OVs to induce pyroptosis has garnered significant attention as a means to enhance their therapeutic efficacy, particularly in breast cancer, a classically immunologically “cold” tumor that OVs can help convert into a “hot” tumor [128]. Recent studies have highlighted that OVs can induce pyroptosis through multiple pathways. Some OVs activate inflammasomes, resulting in caspase-1 activation and GSDMD cleavage [129], whereas others, such as coxsackievirus and parapoxvirus ovis, induce CASP3/GSDME-mediated pyroptosis [66].

In preclinical breast cancer models, OVs have shown promising results in inducing pyroptosis and promoting tumor regression in breast cancer. For example, an oncolytic adenovirus expressing a modified form of TRAIL induces pyroptosis in breast cancer cells, leading to tumor regression and prolonged survival in mouse models [130]. Similarly, an oncolytic vaccinia virus expressing a GSDMD-cleaving protease induced pyroptosis in breast cancer cells, resulting in enhanced antitumor activity [82]. Lin et al. demonstrated that the oncolytic parapoxvirus ovis induces CASP3/GSDME-mediated pyroptosis in EMT6 breast cancer cells [66].

Further breast-specific research has demonstrated that treatment with microvesicle-nanoparticles combined with oncolytic herpes simplex virus 1 results in CASP3/GSDME-mediated tumor cell pyroptosis in a 4T1 TNBC model [66]. This combination also remodels the tumor microenvironment and exhibits synergistic effects with anti-PD-1 therapy [66]. Notably, combining ORFV with chemotherapeutics, such as etoposide, showed increased pyroptotic death and enhanced CD8 + T cell infiltration in a 4T1 TNBC model, with triple therapy (etoposide, ORFV, and anti-PD-1) extending overall survival [66].

Induction of pyroptosis through OVs boosts antitumor immunity, as pyroptotic cell death facilitates immune cell recruitment to the tumor site [66]. Furthermore, cytotoxic lymphocytes, including CAR T-cells, release granzymes that activate GSDMB or GSDME, inducing pyroptosis and enhancing cytotoxicity [87]. The ability of OVs to trigger pyroptosis makes them attractive candidates for combination therapy with immune checkpoint inhibitors and adoptive cell therapy in breast cancer [66].

f. Radiotherapy-Induced Pyroptosis

Radiotherapy, a cornerstone of cancer treatment, exerts antitumor effects by generating ionizing radiation and triggering cell death [56]. Pyroptosis has emerged as a crucial mediator that contributes to both direct tumor cell death and activation of antitumor immunity. Radiation induces DNA damage and oxidative stress, leading to NLRP3 inflammasome activation and gasdermin D (GSDMD)-dependent pyroptosis [56, 81]. Increased serum levels of pyroptosis markers (GSDMD-CT, NLRP3, and IL-18) in patients with breast cancer after radiotherapy confirm the clinical relevance of this pathway [81].

Key studies have demonstrated these mechanisms. Zhang et al. reported radiation-induced NLRP3 inflammasome activation and pyroptosis in breast cancer cells [81], whereas P2Y2R-mediated inflammasome activation has been implicated in radiotherapy-resistant breast cancer [131]. Radiation-induced pyroptosis promotes DAMPs release (HMGB1, IL-1α) and relies on intracellular DNA sensors, such as ZBP1/DAI/DLM-1, enhancing local immune responses [81, 132]. These findings provide a strong rationale for combining radiotherapy with immunotherapy. However, direct evidence linking radiotherapy to pyroptosis in cancer cells remains limited, necessitating further studies [56].

g. Combined Application of Pyroptosis and Cuproptosis

Pyroptosis is a promising therapeutic target for breast cancer, and its efficacy can be enhanced by combining it with other regulated cell death (RCD) pathways, such as cuproptosis. Cuproptosis, a newly discovered RCD mechanism, offers potential for combination therapies through its distinct action and selective toxicity in breast cancer, where copper dysregulation drives progression [133].

i. Cuproptosis as a Therapeutic Strategy

Cuproptosis is a novel copper-dependent form of RCD that is distinct from other well-characterized cell death pathways [134]. It is triggered by the accumulation of copper ions within the mitochondria, which bind directly to and induce the aggregation of lipoylated enzymes in the tricarboxylic acid cycle, such as dihydrolipoamide S-acetyltransferase. This leads to proteotoxic stress, destabilization of iron-sulfur clusters, and ultimately, cell death [135]. Cancer cells often exhibit altered copper metabolism and heightened dependency on copper for proliferation and survival compared to normal cells, making them particularly vulnerable to copper-induced cytotoxicity [133]. This metabolic vulnerability makes cuproptosis an attractive strategy for selective cancer cell eradication [136], especially in breast cancer, where aberrations in copper homeostasis are frequently observed and contribute to disease progression [137]. Key regulators include copper transporters, such as SLC31A1 (influx) and ATP7A/B (efflux), with FDX1 mediating Cu²⁺ reduction to Cu⁺ for protein binding [136]. Understanding copper biology in cancer treatment involves discerning when to activate cuproptosis and when to suppress cuproplasia [138].

ii. Rationale for Combined Pyroptosis-Cuproptosis Induction

The combination of pyroptosis and cuproptosis offers a compelling therapeutic strategy because of their complementary mechanisms and potential for synergistic antitumor effects [139]. Molecularly, cuproptosis is triggered by copper overload binding to lipoylated TCA cycle enzymes, causing protein aggregation, iron-sulfur cluster loss, mitochondrial dysfunction, and ROS burst [133]. This excess copper and ROS amplification crosstalk with pyroptosis by enhancing caspase-3/GSDMD or GSDME cleavage, gasdermin pore formation, and inflammasome activation [140]. Yang et al. rigorously delineated this connection by proteomic interrogation, identifying copper stress-induced dysregulation of mitochondrial proteostasis networks, including upregulated HSPs, peroxiredoxins, and TCA lipoyltransferase subunits that not only destabilize Fe-S clusters to execute cuproptosis but also prime NLRP3 inflammasomes via sustained ROS and lipotoxic stress, forging a bidirectional amplification loop with pyroptotic effectors. This mechanistic convergence, validated through quantitative mass spectrometry and functional rescue assays, underscores the role of copper as a rheostat that toggles metabolic collapse to inflammatory lysis, with heightened relevance in Cu-transport-overexpressing breast tumors [141]. Conversely, pyroptotic pores facilitate ion influx, amplifying mitochondrial stress and creating a feed-forward loop of proteotoxic and inflammatory cell lysis [56]. This intricate interplay suggests that the pharmacological induction of cuproptosis could sensitize breast cancer cells to pyroptotic stimuli, leading to enhanced therapeutic outcomes and potentially overcoming resistance mechanisms often encountered with single-pathway targeting strategies.

What makes this duo special is its orthogonality. Pyroptosis drives pro-inflammatory immunogenic cell death (ICD) via IL-1β/IL-18 release and DAMPs [77], while cuproptosis exploits cancer-specific Cu dependency for metabolic targeting, yielding multi-modal death resistant to single-pathway evasion [139]. Synergy amplifies DAMPs/TAA release, cGAS-STING, DC maturation, and CD8⁺ T infiltration, converting “cold” tumors “hot” [142].

Clinically relevant for breast cancer, preclinical models show 70-90% regression via Cu nanocarriers co-inducing both, synergizing with ICIs/PTT [140, 143]. DSF/Cu links Cu overload to pyroptosis-ICD, promising translation despite the toxicity challenges [144, 145].

iii. Cutting-Edge Research in Combined Pyroptosis-Cuproptosis for Breast Cancer

Recent preclinical investigations have unveiled a spectrum of innovative nanomedicine platforms that co-induce pyroptosis and cuproptosis, yielding synergistic antitumor effects in breast cancer models, particularly TNBC, which is characterized by copper dysregulation, immunosuppressive tumor microenvironments, and resistance to conventional therapies [136, 142, 146]. These strategies exploit copper overload to trigger cuproptosis through mitochondrial lipoylated protein (e.g., DLAT) aggregation and Fe-S cluster depletion [134], while amplifying pyroptosis via ROS-mediated gasdermin (GSDMD/GSDME) cleavage and inflammasome activation [140].

Pioneering H₂S-activated ion-interference therapy by Zhao et al. exemplifies TME-responsive copper orchestration: tumor-derived H₂S triggers nanoparticle disassembly, unleashing Cu²⁺ overload that concurrently drives cuproptotic TCA cycle disruption and pyroptotic GSDMD pore formation, modulating immunosuppression, boosting immune infiltration, and achieving robust efficacy in copper-vulnerable breast cancer [147]. Complementing this, Qiao et al.‘s self-destructive copper carriers disintegrated in acidic TMEs, liberating Cu ions to provoke dual RCD in dormant/recurrent 4T1 TNBC tumors, amplifying tumor-associated antigen release, T-cell priming, and metastasis inhibition, highlighting their potency against post-treatment relapse [148].

Copper-centric bio-coordination nanoparticles from Cun et al. integrated redox modulation and glycolysis inhibition, synergizing ROS-driven GSDMD cleavage with Cu⁺-dependent proteotoxicity for pyroptosis-cuproptosis induction, yielding superior regression in glycolytic breast cancers, and potentiating immunotherapy [149]. Similarly, Wei et al.‘s NIR-II-responsive Cu-N-doped photocatalysts accumulated in tumors, generating superoxide/ROS under irradiation to cleave GSDMD while inducing DLAT aggregation and Fe-S loss, offering spatiotemporal precision, deep penetration, and ICD in 4T1 models [140]. Guan et al. developed Cu₉S₈@AIPH/PAH/siATP7A/HA nanoplatforms, silencing the efflux pump ATP7A to exacerbate intratumoral Cu²⁺, proteotoxic stress, pyroptosis synergy, CD8⁺ influx, and lung metastasis suppression in TNBC orthotopics [142].

Mitochondria-homing innovations include Peng et al.‘s traceable pyruvate-Cu nanoparticles, which disrupt TCA cycles for cuproptosis while synergizing pyroptotic ROS bursts, prolonging survival in TNBC xenografts [150], and Liang et al.‘s Cys/GSH-responsive T-T@Cu, where NIR-II mild photothermal ferroptosis downregulates ATP7A/B, amplifying cuproptosis-pyroptosis in 4T1 models [143]. Guo et al. paired DSF/Cu paradigms with docetaxel for ICD-linked dual RCD, DC maturation, and 4T1 metastasis control [144], while intratumoral DSF/Cu with irradiation boosts GSDMD pores, abscopal effects, and cGAS-STING via mtDNA release [151].

Emerging studies in 2025 extend this paradigm. Xu et al.‘s pH-responsive “copper bomb” self-accelerates via TME acidity/H₂O_2_/GSH fueling Cu catalysis, ATP7B knockdown, and cuproptosis, eliciting innate/adaptive immunity (cGAS-STING/ICD) to reverse TNBC immunosuppression and metastasis [152]. Li et al.‘s mitochondria-targeted CuET nanoparticles amplify immunogenic cuproptosis, polarize TAMs, and inhibit TNBC growth without toxicity [153, 154]. These converge on multimodal TME reprogramming, with consistent 70–90% tumor inhibition, survival extension, and metastasis ablation across 4T1/orthotopic models [136, 146].

Critically, while mechanistically elegant, leveraging Cu-ROS-inflammasome feed-forward [133, 139] translational hurdles persists: Cu toxicity, heterogeneity in Cu transporters (SLC31A1/ATP7A), and absent breast-specific trials [137]. Nanotechnology mitigates off-targets via responsiveness [155], yet subtype-tailoring (e.g., FDX1-low luminal resistance [136]) and combinations with ICIs warrant Phase I exploration, positioning pyroptosis-cuproptosis as a paradigm-shifting axis for immunogenic TNBC therapy [139, 156].

These preclinical studies (e.g., 4T1 and MCF-7/MDA-MB-231 xenografts) consistently reported 70–90% tumor inhibition, extended survival, and TME remodeling [146]. Copper ionophores, such as elesclomol and DSF, show promise; elesclomol synergizes with PARP inhibitors in BRCA-mutant models [136], while DSF/Cu is in Phase II trials for other cancers with preclinical breast efficacy [145]. No dedicated clinical trials for pyroptotic-cuproptotic combinations exist yet, but ongoing DSF/elesclomol studies support translation, addressing subtype heterogeneity via nanotechnology [136, 155].

### Advantages, disadvantages, and clinical translation of pyroptosis-inducing strategies

#### Chemotherapy

a. Advantages: Induces pyroptosis via the caspase-3/GSDME pathway, enhancing immunogenicity and antitumor immunity. This is a well-established therapeutic modality in clinical practice [56]. b. Disadvantages: Reduced GSDME expression in breast cancer limits its therapeutic efficacy. There is a potential for off-target toxicity and immune cell damage [56]. c. Clinical Potential: Restoring GSDME expression using demethylating agents or nanocarrier systems shows promise for improved clinical outcomes [53, 157].

#### Small molecules

a. Advantages: Can target specific pyroptotic pathways, allowing oral/intravenous administration and potentially tumor-selective designs [34]. b. Disadvantages: Limited clinical data exist, with the risk of systemic inflammation if tumor specificity is not achieved [46]. c. Clinical Potential: Considerable potential for future clinical translation, particularly when integrated with targeted delivery systems [34].

#### Immunotherapy

a. Advantages: Pyroptosis bridges innate and adaptive immunity, thereby enhancing the effectiveness of checkpoint inhibitors and CAR-T therapy. This facilitates the conversion of “cold” tumors to “hot” tumors [34]. b. Disadvantages: The potential for excessive inflammation and tumor heterogeneity may impede consistent responses [34]. c. Clinical Potential: Strong scientific rationale for combining pyroptosis-inducing agents with immunotherapies to improve the efficacy of breast cancer treatment [34].

#### Nanotechnology

a. Advantages: Facilitates targeted delivery, controlled release, and tumor-specific activation of pyroptosis, reducing systemic toxicity and amplifying the immune response [53]. b. Disadvantages: Translational challenges persist with possible off-target effects, and long-term safety assessments are required [158]. c. Clinical Potential: Numerous preclinical models have demonstrated efficacy in clinical applications [158].

#### Oncolytic viruses

a. Advantages: Synergistic with nanomedicine, improving pyroptosis induction and antitumor immunity while enhancing tumor penetration [128]. b. Disadvantages: Concerns regarding immune escape and safety remain, and clinical translation remains limited [128]. c. Clinical Potential: Significant promise when used in combination with nanotechnology and immunotherapy for breast cancer treatment [128].

#### Radiotherapy

a. Advantages: Induces pyroptosis and immunogenic cell death; efficacy can be enhanced using radiosensitizers. Widely adopted clinical treatment [81]. b. Disadvantages: Risk of damage to normal tissues; direct evidence of pyroptosis induction in clinical settings is limited [81]. c. Clinical Potential: Combination of nanotechnology and epigenetic drugs can promote apoptosis-to-pyroptosis conversion, improving therapeutic outcomes [159].

### Clinical combination feasibility

Most of these technologies can be effectively combined with pyroptosis induction for breast cancer management, offering a robust therapeutic strategy. This is particularly true when employing nanotechnology or epigenetic modulators, which overcome key limitations such as GSDME silencing and off-target effects, thereby enhancing overall treatment efficacy [34].

**Fig. 5:**
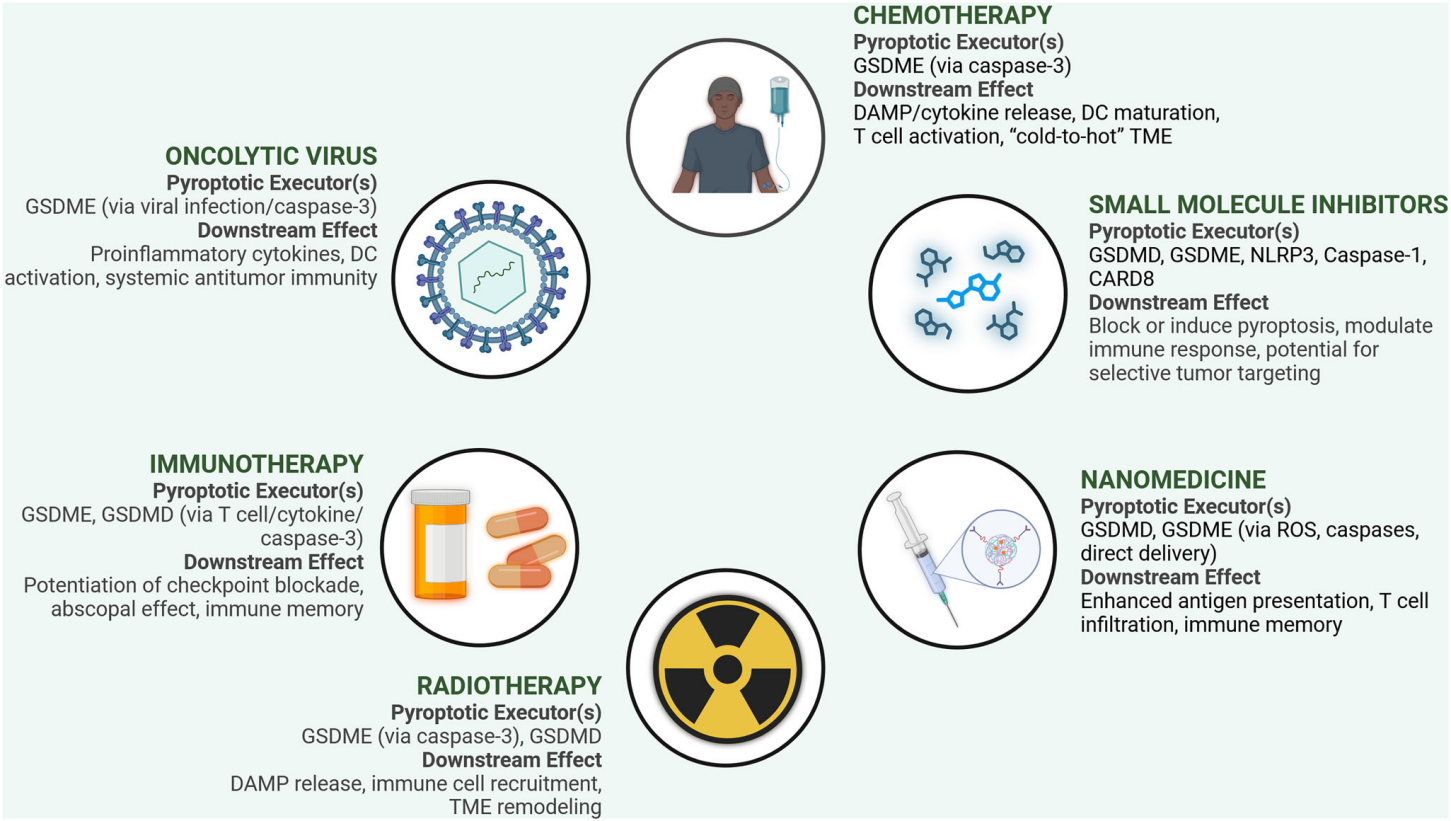
Therapeutic strategies in Breast Cancer therapy. This figure maps major cancer therapies to their pyroptotic executors and their subsequent immune responses. This illustrates how these therapies activate pyroptotic pathways, leading to the release of molecules that trigger robust antitumor immunity. This is achieved by enhancing T cell activation, remodeling the tumor microenvironment, and establishing immune memory. Small-molecule inhibitors are also highlighted for their dual ability to induce or block pyroptosis and modulate the immune checkpoints.

To provide a thorough examination of the existing therapeutic approaches, Fig. 5 synthesizes breast cancer treatments, showing their pyroptotic executors and immune responses in promoting antitumor immunity.

## Clinical correlates and biomarkers of pyroptosis in breast cancer

Research on the clinical applications of pyroptosis is advancing, focusing on identifying markers for patient selection, treatment prediction, and disease monitoring in breast cancer. Key biomarkers include gene signatures, methylation status, circulating GSDMD fragments, and cytokine profiles [160]. Table 4 summarizes the emerging biomarker gene signatures, methylation patterns, circulating GSDMD fragments, and cytokine panels that may predict responsiveness to pyroptosis-modulating therapy.

**Table 4:** Clinical Correlates and Biomarkers of Pyroptosis in Breast Cancer.

Biomarker Type	Key Examples/Signatures	Clinical/Histological Correlates	Ref
Gene Signatures	IL-18, GSDMC, TIRAP (3-gene); CASP9, GPX4, NLRC4, SCAF11, TNF, TIRAP, IL18 (7-gene); 16-gene, 15-gene, 4-gene, 10-gene, 56-gene, lncRNA-based signatures	High-risk scores predict poorer survival, higher recurrence, lower immune infiltration, and distinct immune subtypes	[164]
Serum Markers	IL-1β, IL-18	Elevated levels linked to increased inflammation, tumor progression, and poor prognosis	[42]
Histological Readouts	GSDMC, GSDMD, IL-18, CASP1, NLRP6 (protein expression by IHC); cell swelling, bubble-like protrusions	High GSDMC expression correlates with worse prognosis and increased immune infiltration; decreased CASP1, IL-18, NLRP6 in tumors	[227]
lncRNA Signatures	6-lncRNA, 8-lncRNA, 9-lncRNA, 10-lncRNA, 16-lncRNA signatures	Risk scores based on lncRNAs independently predict prognosis and immune microenvironment status	[228]
Immune Correlates	Immune cell infiltration (CD8 + T, B cells, macrophages), immune checkpoint expression (PD-1, PD-L1, CTLA-4, LAG3)	Low-risk groups show higher immune infiltration and checkpoint expression, indicating better immunotherapy response	[227]

### Prognostic gene signatures

Pyroptosis-related gene signatures have significant prognostic value in breast cancer, offering a robust approach for stratifying patients and predicting clinical outcomes. Numerous studies have developed multi-gene models, validated through comprehensive bioinformatics analyses of public datasets, such as The Cancer Genome Atlas and Gene Expression Omnibus cohorts, to classify patients into distinct risk groups [161–163]. For instance, a three-gene signature effectively identified patients with poor overall survival, progression, and recurrence [42]. Similarly, a seven-gene signature (including CASP9, GPX4, IL18, NLRC4, SCAF11, TIRAP, and TNF) has been rigorously validated for prognostic stratification across different breast cancer cohorts, demonstrating credible predictive capacity with AUC values ranging from 0.775 to 0.806 for 3- to 10-year survival in validation sets such as GSE 20685 [162, 164, 165]. Other models include a five-gene prognostic signature (SEMA3B, IGKC, KLRB1, BIRC3, and PSME2) that exhibited independent prognostic value [68], and a six-gene prognostic signature (GSDMC, IL-18, CHMP3, TP63, GZMB, and CHMP6) that was confirmed as an independent prognostic factor in patients with breast cancer [161]. In TNBC, a six-gene risk model has been shown to effectively predict prognosis [79]. Comprehensive analyses of up to 56 pyroptosis-related genes have further demonstrated significant associations with progression-free survival, disease-specific survival, overall survival, and distinct immune microenvironment characteristics [166]. These investigations revealed that specific pyroptosis-related genes often exhibit frequent mutations and copy number variations, which are critically correlated with immune processes and survival [42, 160, 167]. These signatures are crucial for identifying patients who may benefit most from pyroptosis-modulating therapies, thereby advancing personalized treatment strategies for breast cancer [161].

### Methylation status

Epigenetic regulation, particularly DNA methylation, significantly influences gasdermin gene expression and subsequent pyroptosis in breast cancer. GSDME, which often acts as a tumor suppressor, is frequently silenced via promoter hypermethylation in various cancers, including breast cancer. This silencing represents a key mechanism underlying chemotherapy resistance, as low GSDME expression impairs the antitumor efficacy. Conversely, restoring GSDME expression significantly improves the therapeutic outcome [77].

DNA demethylation, for instance, by agents such as decitabine, can restore GSDME expression and enhance chemosensitivity to drugs such as taxol, thereby converting chemotherapy-induced apoptosis into GSDME-mediated pyroptosis. This suggests that GSDME methylation status is a crucial predictive biomarker for chemotherapy response [168]. The methylation status of GSDME in patients with breast cancer has been linked to lymph node metastases, suggesting its potential to guide the selection of appropriate chemotherapy agents to enhance sensitivity and overcome drug resistance [1].

Modulating GSDME expression using epigenetic drugs, such as decitabine, shows promise in preclinical models for restoring pyroptosis sensitivity in TNBC. Studies have indicated that decitabine can upregulate GSDME expression, shifting cell death from apoptosis to pyroptosis, particularly when GSDME levels are low [46]. However, while decitabine alone may restore GSDME expression, the visible induction of pyroptosis often requires a combination with conventional chemotherapy agents, highlighting GSDME expression as a prerequisite for effective combination therapy [82]. This epigenetic modulation strategy, as seen with HfO_2_ nanoparticles combined with decitabine, successfully converts apoptosis to pyroptosis in TNBC, thereby inhibiting its metastasis [157].

### Circulating GSDMD fragments

Circulating GSDMD fragments, specifically the N-terminal domain, serve as critical indicators of active pyroptosis and function as key executors of this inflammatory cell death pathway. Inflammatory caspases, such as caspases-1, -4, -5, and -11, cleave full-length GSDMD, releasing the GSDMD-N fragment, which oligomerizes to form pores in the cell membrane, ultimately inducing pyroptosis [169].

In breast cancer, particularly in TNBC, elevated serum GSDMD-N levels are robustly correlated with poor overall survival and increased distant metastasis [81]. This suggests that pyroptosis-related inflammation, driven by the release of inflammatory factors orchestrated by GSDMD, actively promotes aggressive disease progression and contributes to unfavorable patient outcomes [170]. Beyond breast cancer, GSDMD has demonstrated broader utility and has emerged as a novel biomarker for early diagnosis in other contexts, such as pleural effusion [171]. Its potential as a pan-biomarker for the early detection and diagnosis of various diseases has been increasingly recognized [172].

Although GSDMD-N holds significant promise as a clinical biomarker, recent preclinical studies have actively explored pyroptotic induction as a therapeutic strategy for breast cancer. For instance, in TNBC models, the cyclin-dependent kinase 2 inhibitor GW-8510 has been shown to triggers GSDME-mediated pyroptosis, which may enhance immune responses. Similarly, Ganoderma lucidum extract and tetraarsenic hexoxide have been shown to induce GSDME-mediated pyroptosis in TNBC cells and mouse models leads to tumor inhibition and reduced metastasis [83]. Chemotherapeutic agents, such as paclitaxel and anthracyclines, commonly used in breast cancer treatment, have also been observed to promote GSDME-mediated pyroptosis and antitumor immunity [60].

To fully realize the clinical potential of GSDMD-N as a biomarker for breast cancer, further rigorous validation is essential. This includes establishing standardized detection methods, such as enzyme-linked immunosorbent assay protocols for GSDMD-CT levels [81], and confirming their consistent clinical utility across diverse patient populations. Future clinical translation of therapeutic strategies targeting pyroptosis will also require extensive investigation to ensure efficacy and safety.

### Cytokine panels

Pyroptosis drives the release of inflammatory cytokines, most notably interleukin-1 beta (IL-1β) and interleukin-18, which serve as crucial biomarkers [173]. Elevated levels of these pro-inflammatory cytokines indicate significant inflammatory activity and pyroptotic cell death in the tumor microenvironment. Although these cytokines can activate antitumor immunity, they also exhibit dual effects. Preclinical studies and clinical observations suggest that IL-1β, despite being identified as a protective factor in some pyroptosis-related gene signatures for TNBC, can promote tumor proliferation and enhance the aggressiveness of TNBC cells by increasing IL-8 and matrix metalloproteinase production [79]. Conversely, blocking IL-1β has been shown to prevent metastasis in a humanized mouse model of breast cancer, and IL-1β antagonists during treatment may even attenuate antitumor adaptive immune responses, implying complex roles [66]. Similarly, IL-18 can suppress breast cancer proliferation and metastasis, indicating antitumor activity [42]. However, increased IL-18 levels are also associated with a poorer prognosis in breast cancer patients, with leptin potentially inducing IL-18 expression in tumor-associated macrophages and breast cancer cells leads to invasion and metastasis [1].

Monitoring these along with other key cytokines such as IL-6, IL-8, and TNF-α, provides critical insights into disease activity, progression, and treatment response [174]. Preclinical studies have shown that the overexpression of IL-6 and IL-8 is associated with tumor progression, metastasis, therapy resistance, and/or poor clinical outcomes in breast cancer. High circulating IL-6 levels are linked to advanced disease, higher recurrence risk, and aggressive phenotypes. Preclinical models have demonstrated that tumor secretion of IL-6 is related to treatment resistance, including tamoxifen resistance in luminal breast cancer and trastuzumab resistance in HER2-positive breast cancer. An ongoing clinical trial is investigating the IL-6 antagonist tocilizumab in combination with trastuzumab and pertuzumab for trastuzumab-resistant metastatic HER2-positive breast cancer [175]. Furthermore, high baseline plasma TNF-α levels were associated with shorter progression-free survival in a phase II study of nivolumab and cabozantinib in metastatic TNBC [176].

Specific cytokine panels offer refined prognostic and predictive capabilities. For example, decreased circulating levels of VEGF, TNF-β, and IL-15 have been associated with PD-L1 overexpression and shorter progression-free survival in patients with primary breast cancer, with low VEGF levels increasing the risk of PD-L1 overexpression [177]. In clinical studies of patients with TNBC receiving neoadjuvant chemotherapy, high levels of IL-13, VEGF, FGF-b, and GM-CSF mRNA were paradoxically associated with worse relapse-free survival, while also being increased in the plasma of patients achieving pathological complete response (pCR), suggesting complex local and systemic immune interactions [178].

Distinct cytokine profiles and pyroptosis-related gene signatures can effectively identify different prognostic groups and patients who might benefit from tailored treatments, even beyond the initial progression, particularly in metastatic breast cancer [179]. Clinical studies evaluating changes in cytokine levels during treatment have identified relationships with clinical outcomes, with longitudinal modifications and cytokine clusters correlating with patient outcomes in patients with metastatic breast cancer treated with eribulin [179]. Time-series analysis of blood cytokine profiles has also shown a correlation with treatment response in patients with TNBC [180]. Nomograms based on serum cytokine-related risk scores have also been developed for breast cancer prognosis [181]. For instance, cisplatin has been shown in preclinical models to induce pyroptosis via the MEG3/NLRP3/caspase-1/GSDMD pathway in TNBC, highlighting potential therapeutic interventions [51]. Overall, these panels provide a comprehensive view of the immune landscape, offering valuable tools for patient stratification and guiding personalized therapeutic strategies in the future.

### Patient selection potential

These biomarkers enable precision-based approaches for patient selection and treatment planning. The integration of gene signatures, methylation status, circulating pyroptotic markers, and cytokine profiles allows clinicians to categorize patients for pyroptosis-inducing therapies, predict treatment response, and monitor outcomes, thereby advancing personalized medicine in breast cancer [167, 182].

## Safety concerns and clinical risk management

Although targeting pyroptosis offers a potent anti-cancer strategy, the inherent inflammatory nature of this cell death pathway necessitates careful consideration of safety and robust clinical risk management [160]. The potential of pyroptosis to trigger systemic inflammation and cytokine release syndrome presents a significant challenge in translating these therapies into clinical practice [183].

### Cytokine release syndrome and systemic inflammation

Pyroptosis is characterized by the release of pro-inflammatory cytokines, including IL-1β and IL-18, and other damage-associated molecular patterns [56]. While crucial for activating antitumor immunity, uncontrolled or widespread pyroptosis can lead to a systemic inflammatory response akin to cytokine release syndrome, a severe and potentially life-threatening complication observed in immunotherapies such as CAR-T cell therapy [184]. Systemic administration of pyroptosis-inducing agents carries the risk of inducing a “cytokine storm,” resulting in multi-organ dysfunction and severe toxicity [185]. Even localized pyroptosis can contribute to systemic inflammation if the released mediators are widely distributed [186]. This emphasizes the need for strategies to balance therapeutic efficacy and mitigate systemic inflammatory adverse events.

### Strategies to balance efficacy with safety

It is paramount to maintain a delicate balance between inducing sufficient pyroptosis for antitumor effects and preventing systemic toxicity. Several strategies have been explored to achieve this goal.

a. Targeted Drug Delivery Systems: Nanotechnology-based drug delivery systems offer a promising avenue for enhancing specificity. Nanoparticles can encapsulate pyroptosis-inducing agents and deliver them preferentially to tumor sites through passive targeting (enhanced permeability and retention effect) or active targeting (ligand binding to cancer cell surface markers). This localized delivery minimizes exposure to healthy tissues, thereby reducing systemic inflammation and off-target effects [111]. Prodrug strategies, in which inactive drugs are activated specifically within the tumor microenvironment, also contribute to enhanced specificity [117].

b. Precision Modulation of Pyroptosis Pathways: The development of highly selective small-molecule inhibitors or activators that specifically target components of the pyroptosis pathway (e.g., GSDMD inhibitors such as disulfiram) can fine-tune pyroptosis [187]. This allows for the controlled induction of pyroptosis in cancer cells while dampening excessive inflammation. However, careful design is required to avoid disrupting essential physiological processes in normal tissues where gasdermin proteins are expressed [187].

c. Combination Therapies with Immunomodulators: Combining pyroptosis-inducing agents with immunomodulators or anti-inflammatory drugs could help manage the inflammatory response. For example, the co-administration of agents that block specific pro-inflammatory cytokines (e.g., IL-1 antagonists such as anakinra) may mitigate CRS-like symptoms while preserving anti-tumor immunity [188].

d. Biomarker-Guided Treatment: Utilizing the aforementioned biomarkers (gene signatures, cytokine panels, and circulating fragments) can help identify patients at a higher risk of adverse events or those who might require closer monitoring or preemptive interventions. Predicting which patients are prone to exaggerated inflammatory responses could enable the development of individualized treatment plans [189].

### Key strategies for clinical risk management

#### Patient sratification and predictive risk models

Recent studies have developed robust pyroptosis-related gene signatures and risk scores to stratify patients based on their prognosis and likely response to therapy. For example, in bladder and breast cancers, high pyroptosis risk scores correlate with increased immune cell infiltration, higher expression of immune checkpoints, greater sensitivity to immunotherapy and chemotherapy, and poorer overall survival [190]. Nomograms that integrate these risk scores with clinical variables (e.g., age and tumor stage) provide clinicians with practical tools for individualized risk assessment and treatment planning [191]. However, these models require further validation in prospective clinical trials and may need to be adjusted for tumor heterogeneity and genetic background [191].

#### Precision targeting and conditional activation

A significant clinical concern is the unintended activation of pyroptosis, which can harm normal tissue and immune cells. To address this, pyroptosis-inducing agents have been modified for conditional activation by disease-specific signals, such as low oxygen levels and tumor biomarker expression. For example, agents activated by hypoxia or acidity and triggered by light allow for precise control over pyroptosis, restricting it to tumor cells and minimizing systemic toxicity [192]. Nanotechnology-based delivery systems further enhance the targeting, solubility, and accumulation of drugs at tumor sites, thereby reducing adverse effects [193].

#### Monitoring and managing inflammatory toxicity

Pyroptosis is inherently inflammatory, increasing the risk of excessive immune activation and tissue damage. Strategies to mitigate these risks include careful patient selection using risk models, monitoring of inflammatory markers, use of agents that modulate inflammasome activity or cytokine release, and combination of pyroptosis-inducing agents with anti-inflammatory or immunomodulatory drugs [46].

#### Overcoming chemoresistance and tumor heterogeneity

Pyroptosis-based treatments can bypass chemotherapy resistance and induce apoptosis. For instance, targeting pathways that do not trigger apoptosis or reactivating pyroptosis in tumors resistant to chemotherapy can restore their sensitivity to drugs [194]. However, differences in the expression of genes related to gasdermin and the functioning of inflammasome components can impact both the effectiveness and risks of treatment, necessitating continuous molecular analyses [95].

#### Biosafety, biocompatibility, and long-term monitoring

The biosafety of novel pyroptotic inducers, particularly nanomaterials, remains a major concern. Although some nanoplatforms show minimal toxicity to healthy tissues owing to selective activation, long-term effects, the potential for immune dysregulation, and unintended effects on normal stem cells remain underexplored. Comprehensive preclinical and clinical toxicity studies and long-term patient monitoring are essential for safe clinical translation [193].

The management of the risks associated with pyroptosis-inducing therapies in clinics involves combining predictive models, precise targeting, inflammation monitoring, and thorough safety assessments to maximize the benefits of treatment while minimizing the harm. Further research is needed to refine these strategies, particularly to develop robust clinical guidelines and novel interventions for managing the potential complications arising from pyroptosis [111, 122].

## Challenges and limitations

Although targeting pyroptosis has tremendous potential for breast cancer treatment, several challenges and limitations need to be addressed to translate this into success.

### Specificity and off-target effects

A key challenge is to ensure that pyroptosis-inducing agent treatments specifically target cancer cells while minimizing their effects on normal tissues [1]. Systemic delivery of these agents can trigger inflammatory responses and tissue damage, thereby limiting their clinical use. Uncontrolled pyroptosis activation in healthy tissues can cause severe side effects, including cytokine storms and organ damage [71]. Clinical trials have struggled to achieve tumor-specific effects without systemic toxicity, and although targeted delivery can enhance specificity, off-target effects persist. To reduce toxicity, researchers have explored delivery systems, such as nanoparticles and antibody-drug conjugates, that selectively target cancer cells [195]. Nanoparticles can accumulate in tumors through enhanced permeability effects or by binding to cancer cell markers [195]. Prodrug strategies can activate drugs specifically within tumors, thereby reducing their systemic toxicity. As cancer cells reduce gasdermin protein expression, while normal cells maintain it, particularly in the gastrointestinal and hematopoietic systems, targeting specificity remains a challenge [66]. The development of selective pyroptosis-inducing agents is essential for minimizing off-target effects and maximizing their efficacy.

### Heterogeneity of breast cancer

Breast cancer comprises multiple subtypes with distinct molecular characteristics that influence the effectiveness of pyroptosis-based therapy [196]. This heterogeneity indicates that subtypes exhibit varying sensitivities to pyroptosis, with genetic modifications affecting key pyroptotic regulators in the tumor cells [77]. Understanding the molecular determinants of pyroptotic sensitivity across subtypes is crucial for patient stratification [197]. Tumors often grow or metastasize after initial stability, as this diversity allows cancer cells to evade therapy and develop drug resistance [198].

Although targeting pyroptosis has shown promise in preclinical studies, the gap between preclinical success and clinical feasibility remains significant owing to breast cancer heterogeneity [196]. Clinical trials (e.g., NCT04852887, NCT03495011, and NCT02476786) have examined intratumoral heterogeneity using precision-based approaches [196]. These trials aim to identify how different subtypes respond to pyroptosis-inducing therapies and to determine the molecular predictors of sensitivity for improved patient stratification.

### Drug resistance

Acquired resistance to pyroptosis-inducing agents may limit their long-term effectiveness [199]. Cancer cells develop resistance by upregulating anti-apoptotic pathways, downregulating pyroptosis proteins, and activating alternative cell death pathways [200]. Combination therapies targeting pyroptosis and resistance mechanisms may improve patient outcomes [201]. Cancer cells undergo Darwinian selection to develop drug-resistant traits at the genomic level. Although precision immunotherapies show promise, resistance remains a significant concern [202]. Targeted therapies face rapid resistance development, requiring approaches to overcome acquired resistance, although combination therapies, such as metformin with targeted drugs, can help in overcoming resistance [199]. Cancer cells can evolve to bypass treatment effects, and dysregulation of signaling pathways affects agent activity [201]. Drug treatment induces cell responses, including growth factor upregulation, promoting tumor repopulation and transition to aggressive phenotypes [202].

Conventional therapies are limited by their lack of cancer cell specificity and high toxicity levels. Resistance leads to tumor recurrence and increased mortality [203]. Resistance mechanisms involve genes, metabolism, inflammation, and neovascularization [204]. The tumor microenvironment mediates tumor-immune cell interactions, contributing to the development of resistance to immunotherapy. These diverse mechanisms necessitate the development of personalized therapeutic strategies based on tumor characteristics of individual patients.

#### Specific mechanisms of drug resistance and potential solutions

The prognosis for patients with advanced-stage breast cancer remains challenging owing to their inherent resistance to conventional treatments, such as radiotherapy and chemotherapy [1]. The specific molecular mechanisms contributing to drug resistance include the intricate regulation of programmed cell death pathways. For instance, despite their pyroptosis-inducing capabilities, widely used chemotherapeutics such as paclitaxel and doxorubicin often encounter rapid resistance in cancer cells [87]. Research has indicated that inhibiting caspase-1 and knocking out GSDMD can induce a taxol-resistant phenotype in cells. The cysteine protease USP47 plays a critical role in doxorubicin resistance, as its ectopic expression leads to doxorubicin resistance, whereas its knockdown increases doxorubicin-induced pyroptosis [87].

The tumor microenvironment significantly contributes to drug resistance in patients with cancer. For example, tumor-associated macrophages act as crucial mediators of cytokine interactions, indirectly influencing the pyroptosis pathway, and playing a key role in resistance within the TME [45]. Furthermore, leptin can induce IL-18 expression in TAMs and breast cancer cells, which subsequently promotes invasion and metastasis, thereby contributing to a pro-tumorigenic environment that facilitates resistance [1].

Several solutions have been proposed to address these resistance mechanisms.

a. Inducing Programmed Cell Death: PCD induction, including pyroptosis, can reverse drug resistance [2]. Novel cell death modalities offer promising avenues for overcoming resistance in breast cancer [205].

b. Targeting Specific Pathways: USP47 knockdown enhances doxorubicin-induced pyroptosis [126]. Tetraarsenic hexoxide induces pyroptosis via the ROS-mediated GSDME pathway, inhibiting tumor growth in TNBC cells [100].

c. Combination Therapies: Natural compounds combined with conventional drugs can address drug resistance [206]. The combination of immunotherapy with pyroptosis-inducing agents modifies the immunosuppressive tumor microenvironment (TME) and restores antitumor immunity [1].

d. Advanced Technologies: AI-powered omics screens for compound pyroptosis drug pairs in TNBC [207]. Precision medicine, which targets the genetic composition of tumors, guides treatment strategies [1]. Identifying pyroptosis-mediated subtypes characterizes TME infiltration and predicts treatment response [208].

### Tumor microenvironment effects

The tumor microenvironment (TME) is crucial for cancer cell survival and therapeutic responses [52]. It comprises tumor cells, immune cells, stromal cells, blood vessels, and non-cellular components, such as the extracellular matrix, cytokines, and exosomes [209]. The TME affects pyroptosis through inflammatory signaling and immune evasion [210]. It contains immunosuppressive cells, such as myeloid-derived suppressor cells (MDSCs) and regulatory T cells, which can modify pyroptosis and reduce antitumor efficacy [211].

Pyroptosis affects antitumor immunity by releasing cytokines, such as IL-1β and IL-18, which have both pro-tumor and tumor-suppressive effects [73]. Targeting TME components while enhancing pyroptosis may improve the antitumor efficacy of therapy. During cancer initiation, immune surveillance enables the recognition of transformed cells; however, as tumors progress, cells escape immune recognition, resulting in treatment resistance [52]. In tumor tissues, cells interact with resident immune and stromal cells, thereby defining tumor progression [212]. These interactions underpin the immune evasion mechanisms and responses to immunotherapy [213]. While immunotherapies, such as checkpoint inhibitors, have improved outcomes, few patients achieve lasting benefits owing to their reliance on pre-existing immunity [56].

Radiotherapy can remodel the tumor matrix and vasculature to enhance the immunotherapy response [214]. The physical characteristics of the TME, including tissue stiffness and fluid pressure, contribute to therapeutic resistance [214]. Targeting the microenvironment through matrix remodeling, angiogenesis inhibition, or immune cell activation can increase pyroptosis and enhance anti-cancer immunity [56, 214]. Normalizing the tumor microenvironment improves antitumor immunity by addressing aberrant vasculature that impedes T cell trafficking [56]. Immunosuppressive cells limit cytotoxic T cell function, whereas other TME components support tumor growth and promote treatment resistance [56, 210].

## Future directions and conclusions

### Future directions

Significant progress has been made in understanding the mechanism of pyroptosis in breast cancer and its implications for therapy. Pyroptosis, a type of programmed cell death that causes inflammation, can both help and harm the tumor. Current research is examining the safety and efficacy of pyroptosis-inducing drugs in various cancers. As we learn more, it is becoming clear that pyroptosis plays a crucial role in the initiation, growth, and therapeutic response of tumors. Efforts to develop treatments that enhance the antitumor effects of pyroptosis while reducing its protumor effects could lead to better outcomes for patients. An important area for future research is the identification of reliable biomarkers to identify patients who are most likely to benefit from pyroptosis-based treatment. For instance, although GSDME levels have shown promise as a predictive marker in some cancers, more research is needed in larger groups of patients with breast cancer. Understanding the molecular mechanisms underlying pyroptosis in cancer is essential for identifying novel targets and pathways. An exciting area of research is the combination of pyroptosis-inducing drugs with immune checkpoint inhibitors to improve antitumor immunity. Pyroptosis can trigger the immune system, potentially making checkpoint inhibitors more effective by affecting the PD-1/PD-L1 axis. Several questions remain regarding which patients are best suited for pyroptosis therapies and how treatment outcomes can be predicted. Therefore, exploring ways to improve targeted drug delivery and combination therapies to prevent resistance and develop more effective treatments for all patients is crucial. Future pyroptosis research and its clinical use will likely focus on the following: a) understanding the molecular mechanisms that control pyroptosis in cancer to identify new targets and pathways; b) developing more powerful and specific pyroptosis-inducing agents; c) improving drug delivery to target tumor cells over normal tissue; d) understanding how the tumor microenvironment and immune system affect pyroptosis and developing combined therapies to overcome immune-induced tumor resistance; e) identifying markers to select patients who will respond best to pyroptosis therapies; and f) conducting well-designed clinical trials to assess the safety and effectiveness of pyroptosis-inducing treatments in a wide range of breast cancer patients. Better methods to check pyroptosis in different cancers are needed to help develop effective treatments that work well. Current tests cannot easily detect pyroptosis in the body; therefore, new methods are needed to visualize this type of cell death non-invasively.

## Conclusion

In conclusion, this review highlights the promising but still emerging understanding of pyroptosis in breast cancer treatment, recognizing its potential beneficial and harmful effects depending on the context. Pyroptosis appears to be an important regulator of cancer, influencing cancer cells and the tumor microenvironment, and impacting tumor growth, metastasis, and treatment response. Inducing pyroptosis in cancer cells may enhance anti-tumor immunity by releasing tumor-associated molecules and inflammatory signals that recruit and activate immune cells. Targeting pyroptosis is gaining attention as a potential therapeutic strategy, either alone or in combination with other treatments. However, a comprehensive and clinically validated understanding of pyroptosis mechanisms and their interaction with the immune system is still developing. It is also important to consider that pyroptosis may contribute to tumor progression and metastasis by creating a pro-tumor microenvironment, underscoring the complexity of its role as a “double-edged sword.” Future research should focus on elucidating the precise biological mechanisms and clinical relevance of pyroptosis in breast cancer, including its influence on the PD-1/PD-L1 axis and tumor microenvironment. Exploring selective modulators of pyroptosis could offer novel therapeutic opportunities, but careful evaluation is necessary to balance its dual effects and optimize patient outcomes.
